# Subunit-dependent and subunit-independent rules of AMPA receptor trafficking during chemical long-term depression in hippocampal neurons

**DOI:** 10.1016/j.jbc.2021.100949

**Published:** 2021-07-10

**Authors:** Shinji Matsuda, Michisuke Yuzaki

**Affiliations:** 1 Department of Engineering Science, Graduate School of Informatics and Engineering, The University of Electro-Communications, Tokyo, Japan; 2 Center for Neuroscience and Biomedical Engineering (CNBE), The University of Electro-Communications, Tokyo, Japan; 3 Department of Physiology, Keio University School of Medicine, Tokyo, Japan

**Keywords:** AMPA, adaptor protein, endocytosis, ionotropic glutamate receptor, LTP, LTD, neuron, phosphorylation, receptor recycling, synaptic plasticity, μ2μ subunit of, AP-2, μ3μ subunit of, AP-3, AMPARs, AMPA-type glutamate receptors, AP, adaptor protein, CaMKII, calmodulin-dependent protein kinase II, EGFP, enhanced green fluorescent protein, GluA1^wt^, WT GluA1, HA, hemagglutinin, HEK293, human embryonic kidney 293, HEK293T, human embryonic kidney 293T, IgG, immunoglobulin G, LTD, long-term depression, LTP, long-term potentiation, MPR, membrane-proximal region, NMDAR, N-methyl-d-aspartate receptor, PSD95, postsynaptic density 95, STG, stargazin, TARPs, transmembrane AMPAR regulatory proteins

## Abstract

Long-term potentiation (LTP) and long-term depression (LTD) of excitatory neurotransmission are believed to be the neuronal basis of learning and memory. Both processes are primarily mediated by neuronal activity–induced transport of postsynaptic AMPA-type glutamate receptors (AMPARs). While AMPAR subunits and their specific phosphorylation sites mediate differential AMPAR trafficking, LTP and LTD could also occur in a subunit-independent manner. Thus, it remains unclear whether and how certain AMPAR subunits with phosphorylation sites are preferentially recruited to or removed from synapses during LTP and LTD. Using immunoblot and immunocytochemical analysis, we show that phosphomimetic mutations of the membrane-proximal region (MPR) in GluA1 AMPAR subunits affect the subunit-dependent endosomal transport of AMPARs during chemical LTD. AP-2 and AP-3, adaptor protein complexes necessary for clathrin-mediated endocytosis and late endosomal/lysosomal trafficking, respectively, are reported to be recruited to AMPARs by binding to the AMPAR auxiliary subunit, stargazin (STG), in an AMPAR subunit–independent manner. However, the association of AP-3, but not AP-2, with STG was indirectly inhibited by the phosphomimetic mutation in the MPR of GluA1. Thus, although AMPARs containing the phosphomimetic mutation at the MPR of GluA1 were endocytosed by a chemical LTD-inducing stimulus, they were quickly recycled back to the cell surface in hippocampal neurons. These results could explain how the phosphorylation status of GluA1-MPR plays a dominant role in subunit-independent STG-mediated AMPAR trafficking during LTD.

Long-term potentiation (LTP) and long-term depression (LTD) of excitatory neurotransmission at glutamatergic synapses have been intensively studied as the neural basis of learning and memory (1, 2). LTP and LTD are mainly caused by changes in the number of postsynaptic AMPA-type glutamate receptors (AMPARs) through activity-dependent lateral diffusion of AMPARs from or to postsynaptic sites, coupled with endosomal transport of AMPARs by exocytosis or endocytosis (3, 4). GluA1 and GluA4 AMPAR subunits are primarily recruited to synapses in an activity-dependent manner (5, 6) during LTP. In contrast, N-methyl-d-aspartate receptor (NMDAR) activation was shown to preferentially induce endocytosis of GluA2-containing AMPARs, followed by subsequent transport to the late endosome/lysosome pathway during LTD (7). In contrast, GluA2-lacking AMPARs are recycled back to the cell surface (7). Indeed, LTD is impaired in the cerebellum lacking GluA2 expression (8). Furthermore, phosphorylation of the GluA1 C terminus by calcium/calmodulin-dependent protein kinase II (CaMKII; Ser831) and PKA (Ser845) has been shown to regulate LTP and LTD (9, 10). Phosphorylation at Ser818 by PKC and phosphomimetic mutation at Ser816 were shown to promote synaptic incorporation of GluA1 (11, 12) (Fig. 1*A*). These findings indicate that activity-dependent AMPAR trafficking is determined by the C terminus of GluA subunits. However, such subunit-specific “rules” have been challenged by recent findings that LTP (13) and LTD (14) do not require the C termini of GluA subunits.

**Figure 1:**
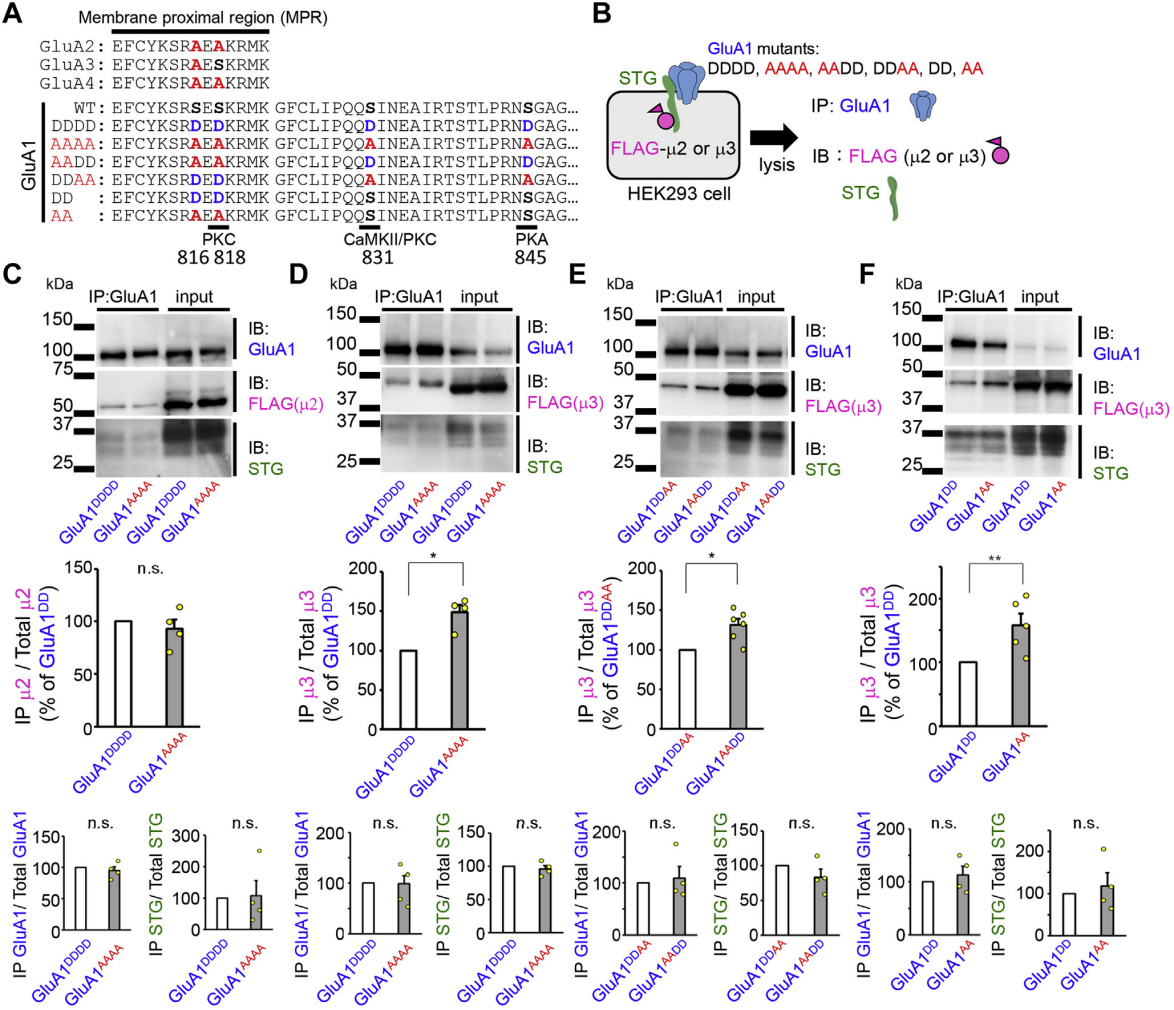
**Phosphomimetic mutations of the MPR regulate the affinity of the AMPA receptor–TARP complex to AP-3.***A*, amino acid sequences of the C terminus of AMPAR subunits and GluA1 mutants. Serine residues that can be phosphorylated by PKC, CaMKII, and PKA are indicated. These residues were replaced with aspartate and alanine to mimic phosphorylation (*blue*) and dephosphorylation (*red*). Although Ser816 is not directly phosphorylated, it enhances the effect of the Ser818 mutation. *B*, schematic drawing of the coimmunoprecipitation assay. Lysates of HEK293 cells expressing STG, GluA1 mutants, and FLAG-tagged μ2 or μ3 were immunoprecipitated using the anti-GluA1 antibody. *C* and *D*, the effect of mutation of all serine residues of GluA1 on the interaction with μ2 or μ3. While μ2 was similarly coimmunoprecipitated with GluA1^AAAA^ and GluA1^DDDD^ (*C*), μ3 was preferentially coimmunoprecipitated with GluA1^AAAA^ than GluA1^DDDD^ (*D*). *Top*, the intensity of the band corresponding to μ2 or μ3 that was coimmunoprecipitated was normalized to the intensity of the respective molecule in the input lysate. Data are presented as the mean + SEM and individual data points (*yellow circles*) (Mann–Whitney U test, ∗*p* < 0.05; n = 4). *Bottom*, the intensity of the band corresponding to GluA1 (*left*) or STG (*right*) coimmunoprecipitated was normalized to the intensity of the respective molecule in the input lysate. Data are presented as the mean + SEM and individual data points. *E*, the effect of the position of the mutations on the interaction with μ3. μ3 was preferentially coimmunoprecipitated with GluA1^AADD^ than with GluA1^DDAA^. The intensity of μ3 in the immunoprecipitated fraction was normalized to that of the input lysate. Data are presented as the mean + SEM and individual data points (Mann–Whitney U-test, ∗*p* < 0.05; n = 6). *F*, the effect of mutations in the MPR on the interaction with μ3. μ3 was preferentially coimmunoprecipitated with GluA1^AA^ than with GluA1^DD^. The intensity of μ3 in the immunoprecipitated fraction was normalized to that of the input lysate. Data are presented as the mean + SEM and individual data points (Mann–Whitney U-test, ∗∗*p* < 0.01; n = 5). μ2, μ subunit of AP-2; μ3, μ subunit of AP-3; AMPARs, AMPA-type glutamate receptors; AP, adaptor protein; CaMKII, calmodulin-dependent protein kinase II; HEK293, human embryonic kidney 293; MPR, membrane-proximal region; n.s., not significant; STG, stargazin; TARPs, transmembrane AMPAR regulatory proteins.

An alternative hypothesis is that AMPAR trafficking is regulated by its auxiliary subunits, such as transmembrane AMPAR regulatory proteins (TARPs), which bind to all AMPAR subunits indiscriminately. The C termini of TARPs stabilize postsynaptic AMPARs by binding to anchoring proteins, such as postsynaptic density 95 (PSD95) (15). The C terminus of TARPs, such as γ-2 (stargazin [STG]), γ-3, and γ-8, contains multiple conserved phosphorylation sites for CaMKII, PKC, and PKA, and positively charged residues (16). Phosphorylation of the C termini of STG is required for hippocampal LTP by enhancing its binding to PSD95 (17). Conversely, the C terminus of STG is dephosphorylated by various chemical LTD-induction protocols in cultured hippocampal (16, 18) and cerebellar (19) neurons. Furthermore, dephosphorylation of STG is required for NMDAR-dependent hippocampal LTD (16, 18) and mGluR1-dependent cerebellar LTD (19) in slice preparations. We previously showed that dephosphorylated TARPs specifically interacted with the μ subunit of the adaptor protein (AP)-2 (μ2) and AP-3 (μ3), which are essential for clathrin-dependent endocytosis and late endosomal/lysosomal trafficking, respectively (18). Thus, activity-dependent phosphorylation status of TARPs during LTP/LTD could affect lateral diffusion of postsynaptic AMPARs, followed by their endocytosis, in a manner independent of AMPAR subunits.

Recently, using mouse lines in which the endogenous C termini of GluA1 and GluA2 were replaced with each other, the C termini of GluA1 and GluA2 were shown to be necessary and sufficient for hippocampal LTP and LTD, respectively (20). Thus, we hypothesized that AMPAR subunits and their phosphorylation status were mechanistically linked with TARP-mediated trafficking. In the present study, we examined whether and how the phosphorylation of GluA1 C terminus could affect its association with STG, a prototype of TARP, and μ subunits of APs, μ2 and μ3. We show that although the PKC phosphorylation sites of GluA1 do not affect its interaction with STG, phosphorylation of GluA1indirectly inhibits μ3 binding to STG. Unless GluA1 was fully dephosphorylated, NMDA-induced LTD was impaired in hippocampal neurons, indicating that TARP-mediated AMPAR trafficking was affected by a subunit-specific rule.

## Results

### Phosphomimetic mutations of GluA1-MPR affects AP-3 binding to STG

The C terminus of GluA1, but not GluA2, contains three serine residues that can undergo phosphorylation by PKC, CaMKII, and PKA (3, 21) (Fig. 1*A*). To test the hypothesis that the phosphorylation status of GluA1 may affect TARP-mediated AMPAR trafficking, we replaced all four serine residues with aspartate (GluA1^DDDD^) and alanine (GluA1^AAAA^), to mimic phosphorylated and dephosphorylated GluA1, respectively. We coexpressed GluA1 mutants, STG, and FLAG-tagged μ2 or μ3 subunits in human embryonic kidney 293 (HEK293) cells and performed coimmunoprecipitation assays (Fig. 1*B*). The anti-GluA1 antibody immunoprecipitated GluA1^DDDD^ and GluA1^AAAA^ similarly (Fig. 1, *C* and *D*). Although GluA1^DDDD^ and GluA1^AAAA^ coimmunoprecipitated STG similarly, the amount of μ3, but not μ2, that coimmunoprecipitated with GluA1^DDDD^ was lower than that with GluA1^AAAA^ (Fig. 1, *C* and *D*). Preimmune immunoglobulin G (IgG) did not immunoprecipitate GluA1, STG, μ2, or μ3 (Fig. S1). To determine the serine residues responsible, we generated GluA1^AADD^ and GluA1^DDAA^, in which either Ser816/Ser818 or Ser831/Ser845 were replaced with alanine or aspartate, without changing the total number of phosphomimetic sites (Fig. 1*A*). The anti-GluA1 antibody immunoprecipitated GluA1^AADD^ and GluA1^DDAA^ similarly. Although GluA1^AADD^ and GluA1^DDAA^ coimmunoprecipitated STG similarly, the amount of μ3 that was coimmunoprecipitated by GluA1^DDAA^ was lower than that by GluA1^AADD^ (Fig. 1*E*), indicating that phosphorylation at Ser816/Ser818 likely reduced the interaction of STG with μ3. Indeed, GluA1^DD^, in which Ser816/Ser818 was replaced with aspartate, coimmunoprecipitated a significantly smaller amount of μ3 than GluA1^AA^, in which Ser816/Ser818 were replaced with alanine (Fig. 1*F*; *p* = 0.008, n = 5, by Mann–Whitney *U*-test). These results indicate that phosphorylation at the membrane-proximal region (MPR) of GluA1 (Fig. 1*A*) affects μ3 binding to the AMPAR–STG complex.

### GluA1-MPR enhances the interaction between STG and AP-3

**Figure 2:**
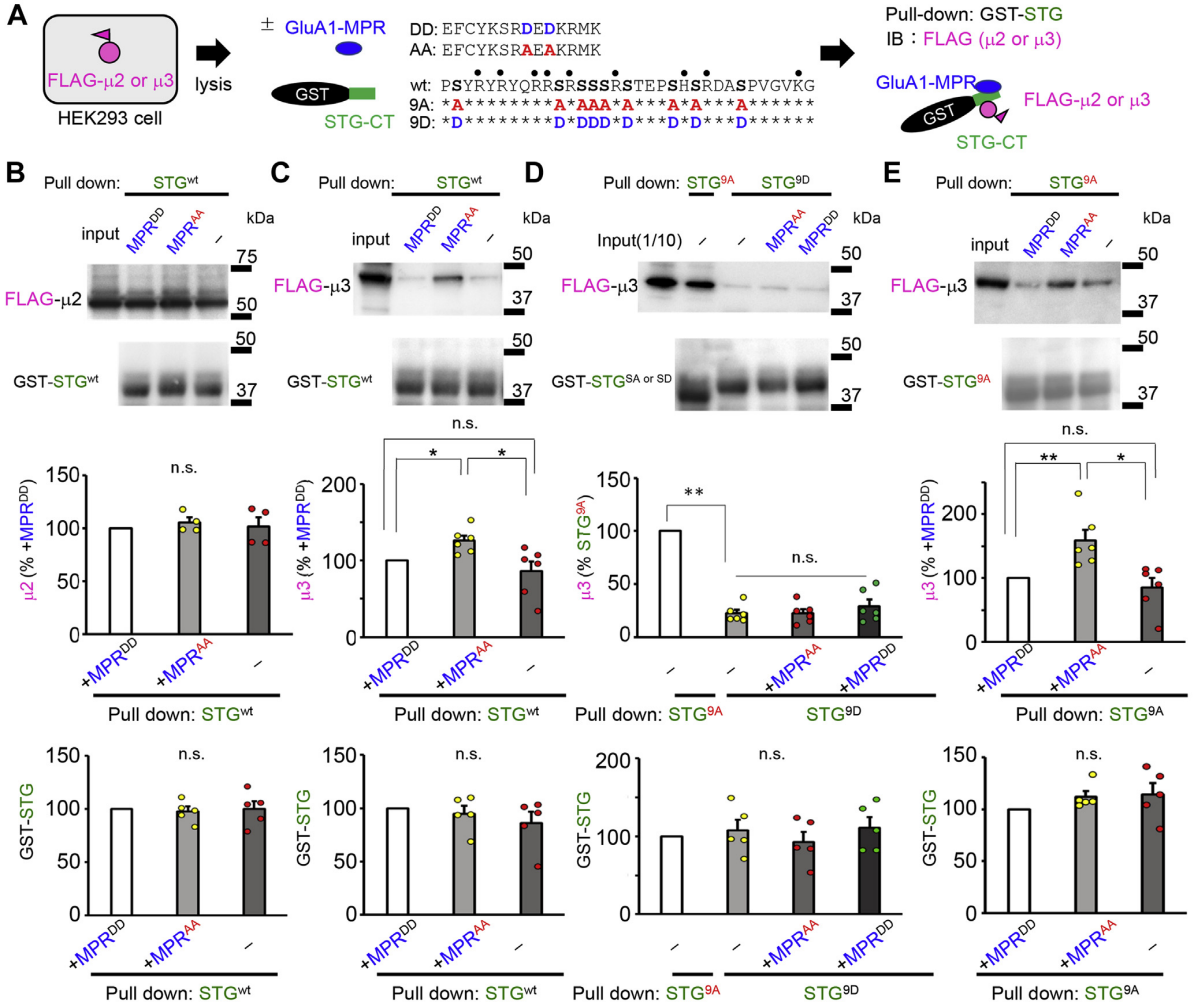
**Phospho-deficient MPR enhances the interaction between STG and AP-3.***A*, schematic drawing of the pull-down assay. Lysates of HEK293 cells expressing FLAG-tagged μ2 or μ3 were pulled down with the GST-fused C terminus of STG (GST-CT) in the presence or absence of synthetic peptides corresponding to the MPR of GluA1. Amino acid sequences of the MPR and STG-CT, in which serine residues were replaced with alanine (*red*) or aspartate (*blue*) to mimic phosphorylated and dephosphorylated forms, are shown. *B* and *C*, pull-down assays showing the effect of the MPR on the interaction between WT STG and μ2 or μ3. *Top*, the amount of μ2 or μ3 that was pulled down with GST-STG^wt^ in the presence of MPR^DD^ was arbitrarily established as 100%. The addition of MPR^DD^ or MPR^AA^ did not affect the interaction between STG^wt^ and μ2 (*B*), whereas MPR^AA^ enhanced the interaction between STG^wt^ and μ3 (*C*). Data are presented as the mean + SEM and individual data points. The Kruskal–Wallis test and Steel–Dwass post hoc test, ∗*p* < 0.05; n = 6 each. *Bottom*, the graphs indicate the amount of pulled down GST-STG. The amount of GST-STG in the pulled-down fraction with MPR^DD^ was arbitrarily established as 100%. *D*, pull-down assays showing the effect of the MPR on the interaction between μ3 and STG^9A^ or STG^9D^. The amount of μ3 that was pulled down with GST-STG^9A^ without the addition of the MPR was arbitrarily established as 100%. Phosphomimetic mutation of STG (STG^9D^) significantly reduced the amount of pulled down μ3. Data are presented as the mean + SEM and individual data points. Mann–Whitney U-test, ∗∗*p* < 0.01; n = 6 each. The MPR peptides did not affect the interaction between μ3 and STG^9D^. Kruskal–Wallis test and Steel–Dwass post hoc test, n = 6 each. *E*, pull-down assays showing the effect of the MPR on the interaction between STG^9A^ and μ3. The amount of μ3 that pulled down with GST-STG^9A^ in the presence of MPR^DD^ was arbitrarily established as 100%. The addition of MPR^AA^ enhanced the interaction between STG^9A^ and μ3. Data are presented as the mean + SEM and individual data points. The Kruskal–Wallis test and Steel–Dwass post hoc test, ∗∗*p* < 0.01 and ∗*p* < 0.05; n = 6 each. μ2, μ subunit of AP-2; μ3, μ subunit of AP-3; AP, adaptor protein; MPR, membrane-proximal region; n.s., not significant; STG, stargazin.

To assess how the MPR of GluA1 affects interaction of μ3 with STG, we prepared the C terminus of STG as a glutathione *S*-transferase (GST) fusion protein and performed pull-down assays using cell lysates of HEK293 cells expressing FLAG-tagged μ2 or μ3. We synthesized the MPR peptide mimicking phosphorylated (MPR^DD^) or unphosphorylated (MPR^AA^) GluA1 and added it to the lysate at a concentration of 500 μM (Fig. 2*A*). The presence of MPR^AA^ or MPR^DD^ did not affect the amount of μ2 pulled down by GST-STG (Fig. 2*B*; n = 4, *p* = 0.99 by the Kruskal–Wallis test). There was no difference in the amount of precipitated GST-STG; however, the amount of μ3 pulled down by GST-STG was significantly increased by the addition of the MPR^AA^ peptide (Fig. 2*C*; MPR^AA^, 126 ± 18%; MPR^DD^, 100%; without MPR, 86 ± 13%; *p* = 0.006, MPR^AA^ *versus* MPR^DD^; *p* = 0.043, MPR^AA^ *versus* −MPR, n = 6 each, by the Kruskal–Wallis test and Steel–Dwass post hoc test). These results indicate that the presence of an unphosphorylated MPR of GluA1 selectively enhanced the interaction between STG and μ3.

STG itself contains multiple positively charged residues and phosphorylation sites at the C terminus (Fig. 2*A*). We next examined whether the facilitatory effect of the MPR^AA^ on STG–μ3 interaction was affected by the phosphorylation status of STG. As reported previously, the amount of μ3 pulled down by GST-STG^9D^, in which nine serine residues were replaced with aspartate to mimic phosphorylated STG, was significantly lower than that pulled down by GST-STG^9A^, mimicking the unphosphorylated form (Fig. 2*D*; STG^9A^, 100%; STG^9D^, 21 ± 3%; *p* = 0.002, n = 6 each, by the Mann–Whitney *U*-test). The presence of MPR^AA^ or MPR^DD^ did not affect the amount of μ3 pulled down by STG^9D^ (Fig. 2*D*; *p* = 0.48; Kruskal–Wallis test). In contrast, the amount of μ3 pulled down by STG^9A^ was significantly increased by the addition of the MPR^AA^ (Fig. 2*E*). These results indicate that the interaction between STG and μ3 is favored when the STG is unphosphorylated and that the presence of unphosphorylated GluA1-MPR further enhances STG–μ3 association.

### GluA1-MPR directly binds STG and indirectly enhances STG–AP-3 interaction

**Figure 3:**
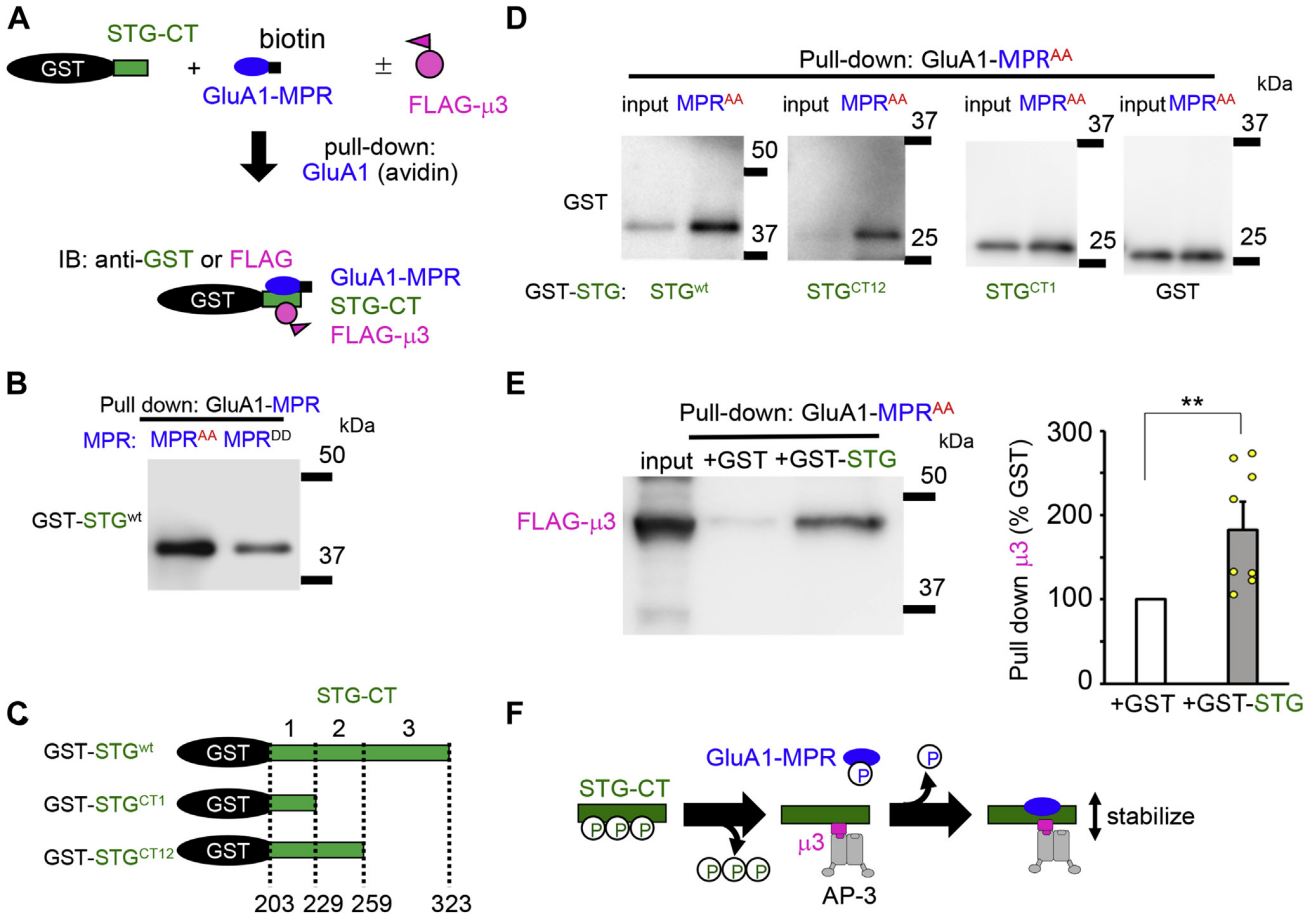
**Phospho-deficient MPR directly binds to the C terminus of STG.***A*, schematic drawing of the pull-down assay. The GST-fused C terminus of STG (STG-CT) was pulled down using avidin that interacted with a synthetic biotinylated MPR peptide. In some experiments, lysates of HEK293 cells expressing FLAG-tagged μ3 were added. *B*, pull-down assays showing a direct interaction between the STG-CT and MPR. Phospho-deficient MPR (MPR^AA^) showed a stronger interaction with the STG-CT than phosphomimetic MPR (MPR^DD^). *C*, schematic drawing of the deletion mutants of the GST-fused C terminus of STG. *Lower numbers* indicate the amino acid position of full-length STG. *D*, pull-down assays showing the interaction between STG deletion mutants and GluA1-MPR. The amount of STG pulled down with GluA1-MPR^AA^ was reduced by the deletion of amino acids 229 to 259 (STG^CT1^). *E*, pull-down assays showing GluA1-MPR indirectly associates with μ3 *via* STG. A larger amount of μ3 was pulled down by GluA-MPR^AA^ when lysates of HEK293 cells expressing FLAG-tagged μ3 were added. The amount of μ3 pulled down with MPR^AA^ in the presence of GST was arbitrary established as 100%. Data are presented as the mean + SEM and individual data points. Mann–Whitney U-test, ∗∗*p* < 0.01; n = 8. *F*, schematic drawing of the enhanced interaction between STG and AP-3 by addition of the dephosphorylated MPR. Dephosphorylated STG can interact with AP-3, and this interaction is further enhanced by the binding of dephosphorylated GluA1-MPR to the CT2 region of STG. MPR, membrane-proximal region; STG, stargazin.

To examine whether and how the MPR of GluA1 binds to the C terminus of STG, we synthesized biotinylated MPR^DD^ and MPR^AA^ and performed a pull-down assay using streptavidin beads (Fig. 3*A*). GluA1-MPR^AA^ pulled down GST-STG much more than MPR^DD^ (Fig. 3*B*). To identify the region of STG necessary for MPR binding, we prepared GST-STG^CT1^ and GST-STG^CT12^, in which the C terminus of STG was sequentially deleted (Fig. 3*C*). Although STG^wt^ and STG^CT12^ were similarly pulled down by GluA1-MPR^AA^, STG^CT1^ was not (Fig. 3*D*), indicating that the CT2 region (230–259) was mediating binding to the MPR of GluA1. When the cell lysates from HEK293 cells expressing FLAG-tagged μ3 were pulled down by biotinylated MPR^AA^ in the presence of GST or GST-STG^wt^ (Fig. 3*A*), a large amount of μ3 was pulled down by MPR^AA^ in the presence of GST-STG^wt^ compared with GST (Fig. 3*E*; GST only, 100%; GST-STG^wt^, 180 ± 34%; *p* = 0.0003, n = 8, by the Mann–Whitney U test), indicating that μ3 indirectly associates with the STG–MPR complex. Together, we propose that dephosphorylated STG directly binds to μ3 and that dephosphorylated GluA1-MPR could further bind to STG and indirectly enhance the GluA1–STG complex (Fig. 3*F*).

### Phosphomimetic mutations of GluA1-MPR regulates NMDA-induced LTD

**Figure 4:**
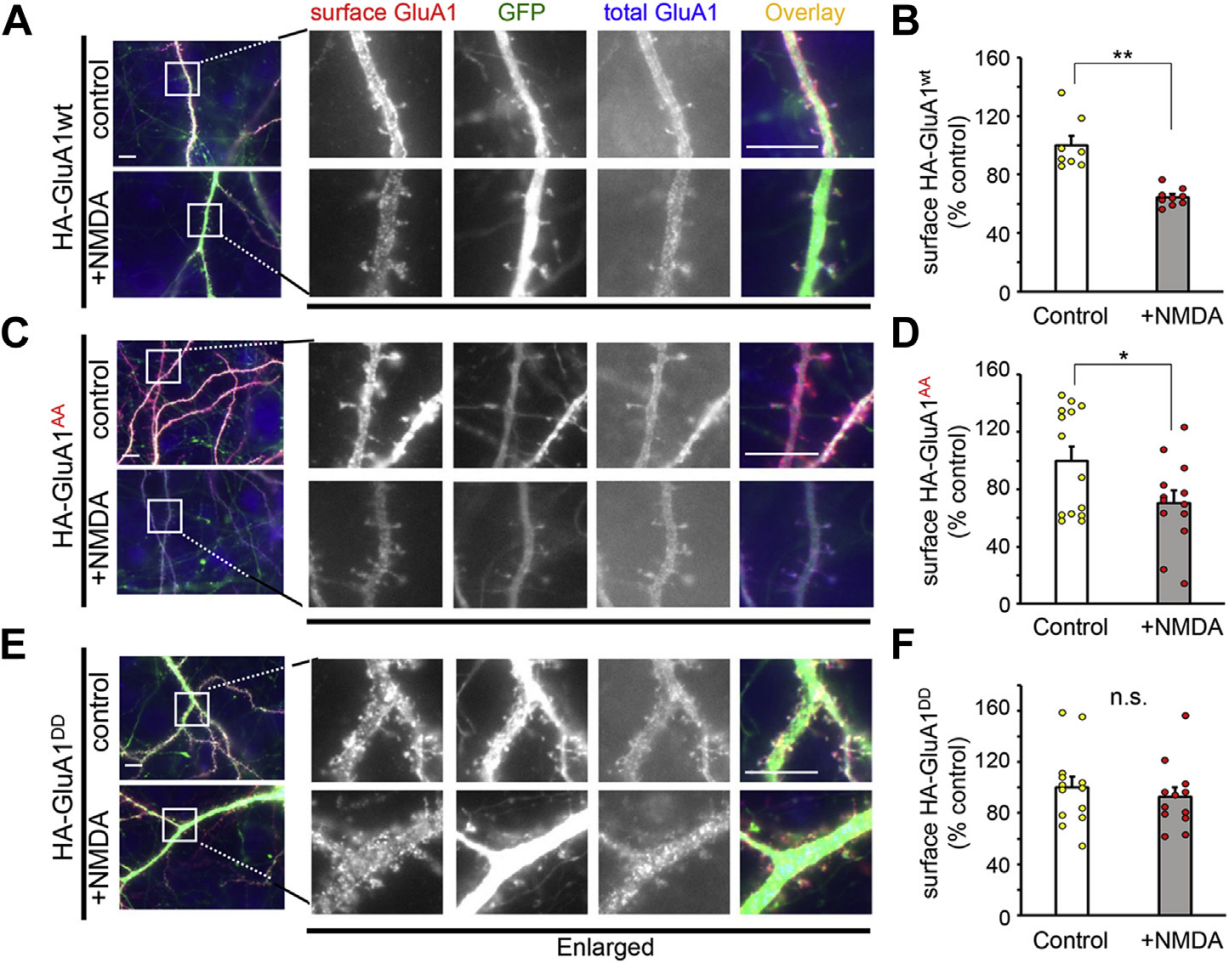
**MPR regulates NMDA-induced AMPAR internalization.***A*, *C*, and *E*, immunocytochemical analysis of the effects of the MPR on NMDA-induced trafficking of cell surface GluA1. Cultured hippocampal neurons expressing enhanced green fluorescent protein and HA-tagged WT (GluA1^wt^) (*A*) or phospho-deficient GluA1 (GluA1^AA^) (*C*) or phosphomimetic GluA1 (GluA1^DD^) (*E*) were treated with 50 μM NMDA for 10 min. Cell surface HA-GluA1 were stained (*red*) after fixation, and neurons were immunostained for total HA-GluA1 (*blue*) after treatment with Triton-X. The dendritic regions marked by *squares* were enlarged in the panels to the *right*. The scale bars represent 10 μm. *B*, *D*, and *F*, quantification of NMDA-induced reduction in the ratio of the surface to total GluA1 fluorescence intensities with NMDA treatment. Data are represented as the ratio of surface HA-GluA1 immunoreactivity normalized by total HA-GluA1 immunoreactivity. The ratio in control neurons was defined as 100% (n = 8–14). Data are presented as the mean + SEM and individual data points. ∗∗*p* < 0.01 and ∗*p* < 0.05; n.s. by two-tailed Student’s *t* test. AMPARs, AMPA-type glutamate receptors; HA, hemagglutinin; MPR, membrane-proximal region; n.s., not significant; NMDA, N-methyl-d-aspartate.

To clarify the role of phosphorylation of GluA1-MPR on AMPAR trafficking, we used a chemical LTD model, in which NMDA application induces AMPAR endocytosis (7, 18). We expressed mutant GluA1, in which a hemagglutinin (HA) tag was added to the N-terminal extracellular domain, and Ser816/Ser818 were replaced with aspartate (GluA1^DD^) or alanine (GluA1^AA^), in cultured hippocampal neurons. After treatment with NMDA (50 μM) for 10 min, the cell surface and total GluA1 were sequentially detected by an anti-HA antibody before and after permeabilizing the plasma membrane (Fig. 4, *A*, *C* and *E*). Mutations in the MPR did not affect the total and surface expression levels of GluA1 at the basal state (Fig. S2). NMDA treatment reduced the intensity of cell surface HA–WT GluA1 (GluA1^wt^) (Fig. 4, *A* and *B*; *p* = 0.0006, n = 8–9 cells) and HA-GluA1^AA^ (Fig. 4, *C* and *D*; *p* = 0.03, n = 13–14 cells, by two-tailed Student’s *t* test). In contrast, the intensity of cell surface HA-GluA1^DD^ was not affected by the NMDA treatment (Fig. 4, *E* and *F*; *p* = 0.53, n = 12–13 cells, by two-tailed Student’s *t* test). These results indicate that phosphorylation of GluA1-MPR inhibits NMDA-induced AMPAR endocytosis during chemical LTD.

### Phosphomimetic mutations of GluA1-MPR regulates trafficking to the late endosome/lysosome

**Figure 5:**
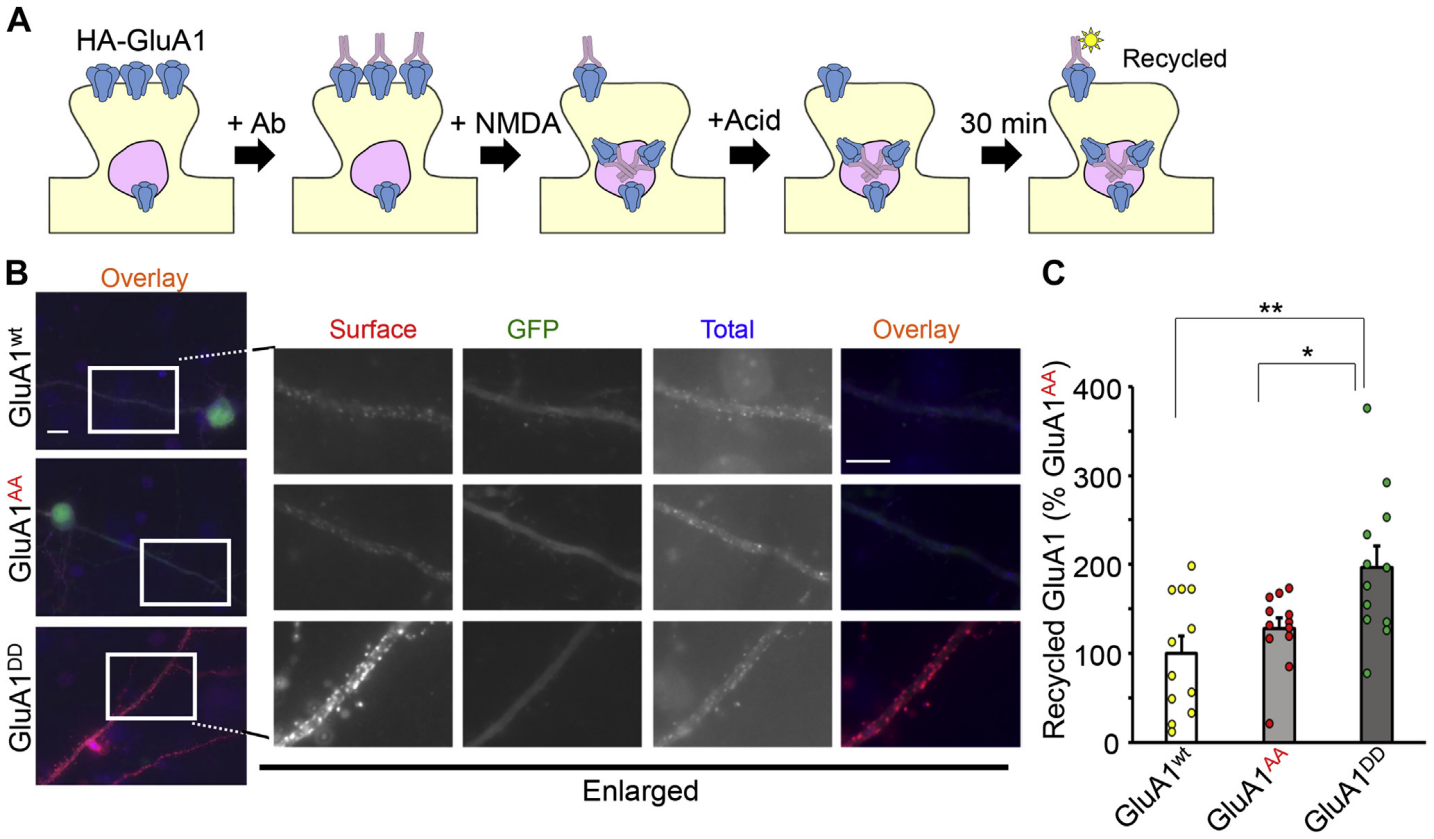
**Phosphomimetic mutations of the MPR increased recycling of GluA1 to the cell surface.***A*, schematic drawing of antibody feeding assay. Living neurons expressing HA-GluA1 mutants were labeled with an anti-HA antibody. After NMDA treatment, remaining cell surface antibodies were removed by acid treatment. After a 30-min incubation to allow the recycling of HA-GluA1, neurons were fixed and recycled, and internal HA-GluA1 was visualized by Alexa 546– and Alexa 350–conjugated secondary antibodies, respectively. *B*, immunocytochemical analysis of the effects of the MPR phosphorylation on the recycling of GluA1 after NMDA treatment. Cultured living hippocampal neurons expressing HA-GluA1^wt^ or HA-GluA1^AA^ or HA-GluA1^DD^ were subjected to the antibody feeding assay. The dendritic regions marked by *white rectangles* are enlarged in the panels to the *right*. The scale bars represent 10 μm. *C*, quantification of the recycled GluA1. Data are represented as the ratio of recycled HA-GluA1 staining/total HA-GluA1 staining intensity. The ratio of HA-GluA1^wt^ was defined as 100% (n = 12 cells). Data are presented as the mean + SEM and individual data points. ∗∗*p* < 0.01 and ∗*p* < 0.05 by one-way ANOVA and Student–Newman–Keuls post hoc test. HA, hemagglutinin; MPR, membrane-proximal region; NMDA, N-methyl-d-aspartate.

The number of cell-surface AMPARs is determined by the balance between endocytosis and exocytosis. To clarify the effect of phosphorylation of GluA1-MPR on AMPAR trafficking, we performed an antibody-feeding assay (18) (Fig. 5*A*). HA-GluA1 on the cell surface of living neurons was first labeled with an anti-HA antibody, and NMDA was applied to the neurons to induce AMPAR endocytosis. After removal of the anti-HA antibody remaining on the cell surface by acid treatment, the population of HA-GluA1 that was endocytosed by the NMDA treatment and recycled to the cell surface within 30 min was specifically visualized. The antibody-feeding assay indicated that the amount of recycled HA-GluA1^DD^ was significantly larger than that of HA-GluA1^wt^ and HA-GluA1^AA^ (Fig. 5, *B* and *C*; HA-GluA1^wt^, 100 ± 19%; HA-GluA1^AA^, 128 ± 12%; HA-GluA1^DD^, 197 ± 24%; *p* = 0.003, HA-GluA1^wt^ *versus* HA-GluA1^DD^; *p* = 0.015, HA-GluA1^AA^ *versus* HA-GluA1^DD^; n = 12 cells each, by one-way ANOVA and the Student-Newman-Keuls post hoc test). These results indicate that although HA-GluA1^DD^ was endocytosed in response to NMDA treatment, it was recycled back to the cell surface.

**Figure 6:**
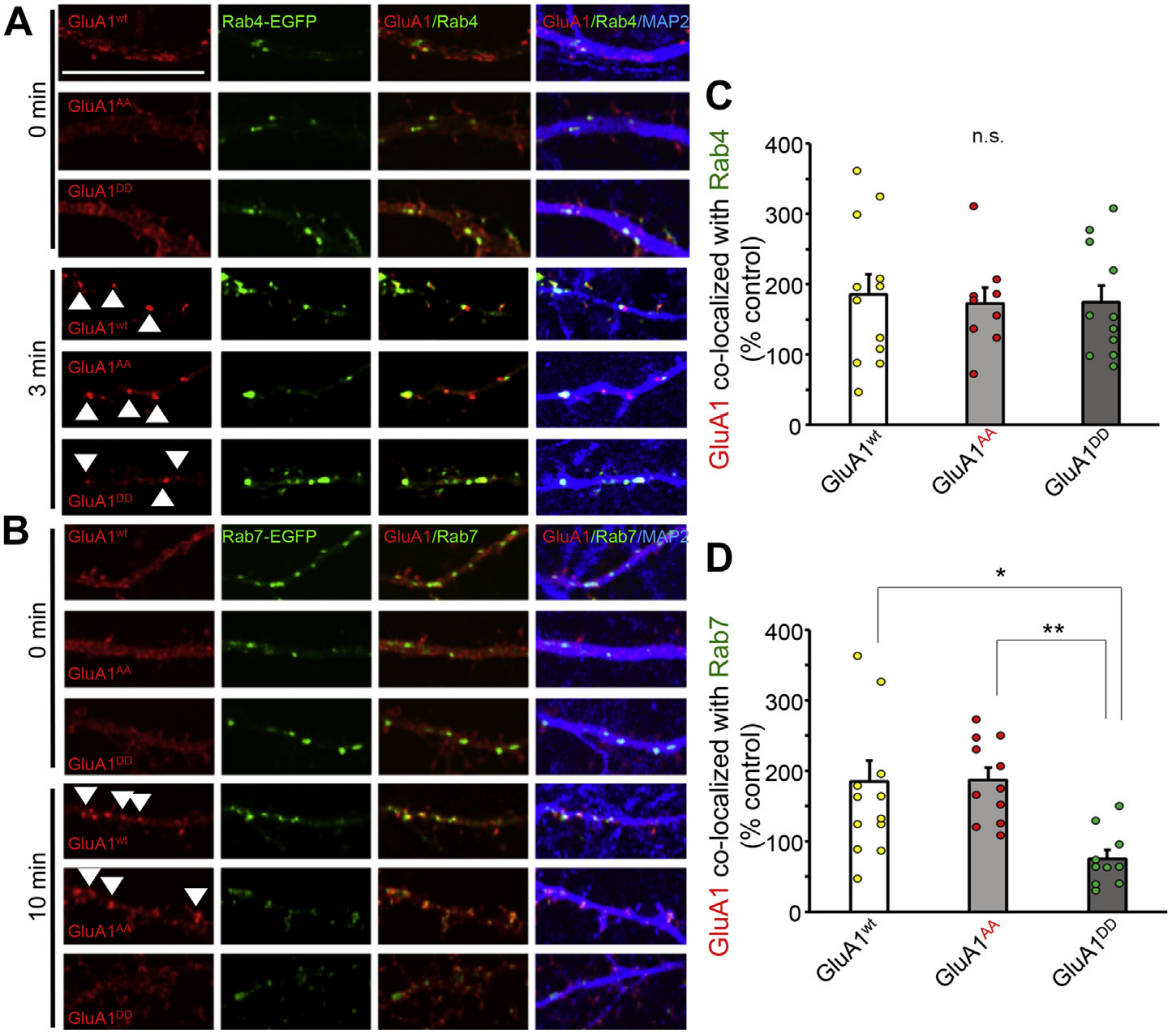
**Phosphomimetic mutations of the MPR inhibit the transport of GluA1 to late endosomes and lysosomes.***A*, colocalization of HA-tagged mutant GluA1 with an early endosome marker, enhanced green fluorescent protein-tagged Rab4 at 0 min, and 3 min after NMDA treatment. The scale bar represents 10 μm. *B*, colocalization of HA-tagged mutant GluA1 with a late endosome/lysosome marker, enhanced green fluorescent protein-tagged Rab7 at 0 min, and 10 min after NMDA treatment. *C* and *D*, quantification of the colocalization of HA-tagged WT or mutant GluA1 with Rab proteins. Data are represented as the ratio of colocalized HA-GluA1 staining/total HA-GluA1 staining intensity. The ratio in the neurons without NMDA stimulation (0 min) was defined as 100% (n = 9–12 cells). Data are presented as the mean + SEM and individual data points. ∗∗*p* < 0.01 and ∗*p* < 0.05; n.s. by the Kruskal–Wallis test and Steel–Dwass post hoc test. HA, hemagglutinin; MPR, membrane-proximal region; n.s., not significant; NMDA, N-methyl-d-aspartate.

To gain mechanistic insight into how phosphorylation of GluA1-MPR affects AMPAR trafficking, we coexpressed HA-GluA1^wt^, HA-GluA1^AA^, or HA-GluA1^DD^ with enhanced green fluorescent protein (EGFP)-tagged Rab4 to label early endosomes in hippocampal neurons. We also used EGFP-Rab7 to detect late endosomes or/and lysosomes and immunostained MAP2 to identify dendrites. HA-GluA1^wt^ and HA-GluA1^AA^ immunoreactivities were colocalized with Rab4 at 3 min, and Rab7 at 10 min along dendrites after NMDA treatment (Fig. 6, *A* and *B*). In contrast, although HA-GluA1^DD^ immunoreactivity was colocalized with Rab4 at 3 min, it did not overlap with Rab7 at 10 min after NMDA treatment (Fig. 6, *A* and *B*). Quantitative analysis indicated that HA-GluA1^wt^, HA-GluA1^DD^, and HA-GluA1^AA^ were similarly colocalized with Rab4 at 3 min after NMDA treatment (Fig. 6*C*; n = 9–12 cells, *p* = 0.95 by the Kruskal–Wallis test). In addition, HA-GluA1DD showed significantly lower levels of colocalization with Rab7 than HA-GluA1^wt^ and HA-GluA1^AA^ at 10 min after NMDA treatment (Fig. 6*D*; *p* = 0.021, HA-GluA1^wt^ *versus* HA-GluA1^DD^; *p* = 0.001, HA-GluA1^AA^ *versus* HA-GluA1^DD^; n = 10–12 cells each, by the Kruskal–Wallis test and Steel–Dwass post hoc test). These results indicate that phosphorylation of GluA1-MPR regulates NMDA-induced AMPAR endocytosis by controlling the transport of AMPARs from early endosomes to late endosomes/lysosomes.

### Interaction among multiple phosphorylation sites at the GluA1 C terminus

**Figure 7:**
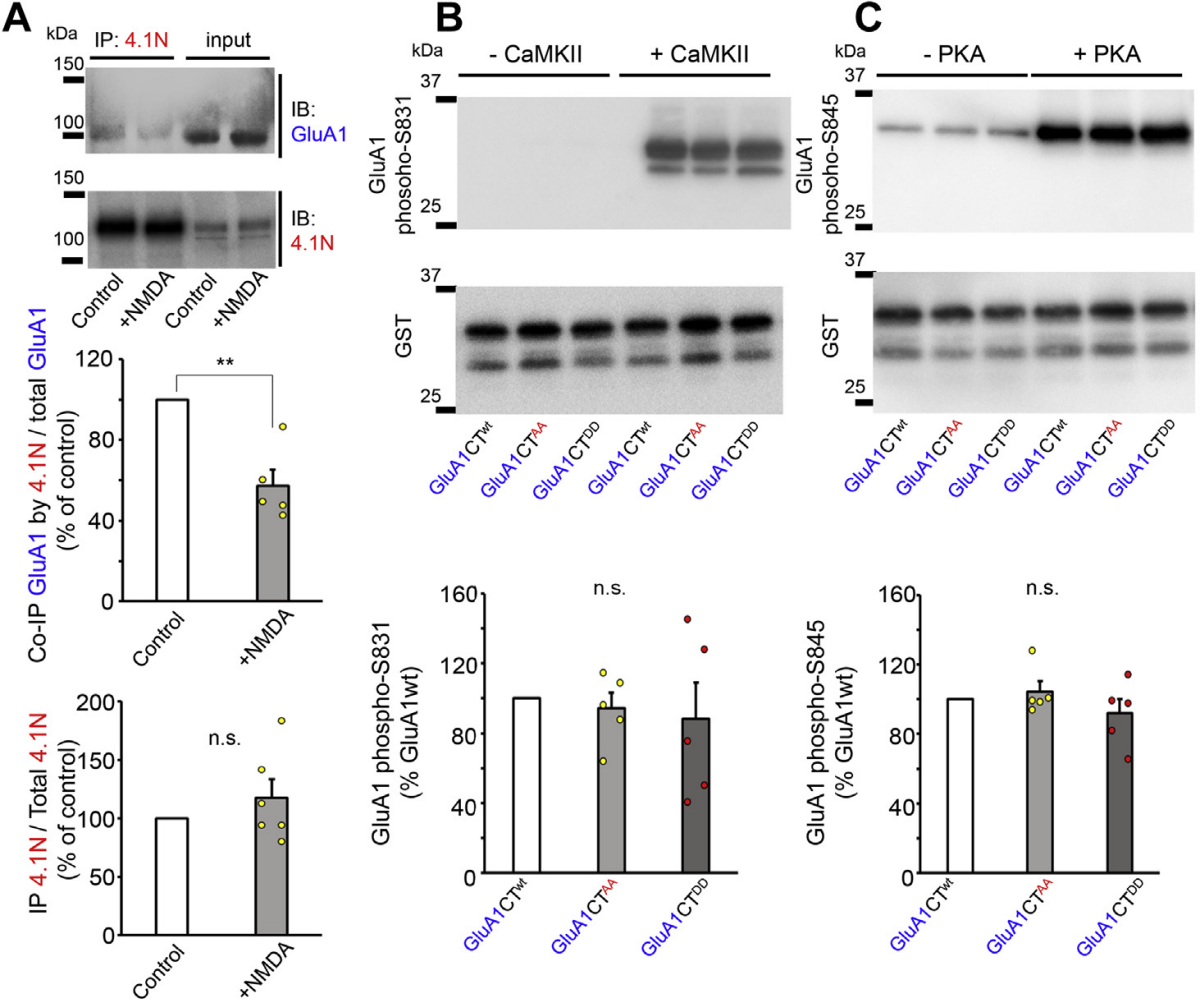
**Phosphorylation of GluA1 MPR, Seri831, and Ser845.***A*, phosphorylation of the GluA1 MPR by NMDA stimulation. Cultured hippocampal neurons were treated with 50 μM NMDA for 10 min and immunoprecipitated using the anti-4.1N antibody after solubilization. The amount of immunoprecipitated 4.1N and coimmunoprecipitated GluA1 was analyzed by immunoblot analysis. The intensities of the bands corresponding to GluA1 (*top*) and 4.1N (*bottom*) in the immunoprecipitated (IPed) fraction were normalized to the intensity of the respective molecule in the input fraction. Data are presented as the mean + SEM and individual data points (Mann–Whitney U test, ∗∗*p* < 0.01; n = 5–6). *B*, Ser831 phosphorylation by CaMKII was not affected by mutations in the MPR. GST fusion proteins with C termini of GluA1^wt^ (GluA1CT^wt^), GluA1^AA^ (GluA1CT^AA^), and GluA1^DD^ (GluA1CT^DD^) were phosphorylated by CaMKII *in vitro* and analyzed by the immunoblot analysis using anti-phospho-Ser831 GluA1 (*top*) and anti-GST (*bottom*) antibodies. Data are presented as the mean + SEM and individual data points (Kruskal–Wallis test; n = 5). *C*, Ser845 phosphorylation by PKA was not affected by mutations in the MPR. GluA1CT^wt^, GluA1CT^AA^, and GluA1CT^DD^ were phosphorylated *in vitro* by PKA and analyzed by immunoblot analysis using anti-phosphor-Ser845 GluA1 and anti-GST antibodies. Data are presented as the mean + SEM and individual data points (Kruskal–Wallis test; n = 5). CaMKII, calmodulin-dependent protein kinase II; MPR, membrane-proximal region; n.s., not significant; NMDA, N-methyl-d-aspartate.

The necessity of PKC phosphorylation at Ser816/Ser818 for LTP expression was demonstrated by enhancing 4.1N binding to GluA1 (12). We immunoprecipitated endogenous 4.1N from the cell lysate of cultured hippocampal neurons to examine whether chemical LTD stimulation affected the interaction between 4.1N and GluA1. We found that the amount of GluA1 coimmunoprecipitated by 4.1N was significantly reduced after NMDA treatment (Fig. 7*A*; n = 5, *p* = 0.008, by the Mann–Whitney U test), whereas preimmune IgG did not precipitate GluA1 or 4.1N (Fig. S1*C*). These results suggest that GluA1 is dephosphorylated at Ser816/Ser818 by chemical LTD induction, and its reduced binding to 4.1N may also contribute to stable LTD expression by reducing reinsertion of AMPARs.

Phosphorylation of GluA1 at Ser831 and Ser845 has been shown to regulate LTP and LTD (9, 10). To examine whether the phosphomimetic or phospho-deficient mutations of GluA1 MPR affected the phosphorylation at Ser831 and 845, we carried out an *in vitro* phosphorylation assay using GST-fused GluA1 C termini. We found that GST fused with the C termini of GluA1^wt^, phospho-deficient GluA1^AA^, and phosphomimetic GluA1^DD^ was phosphorylated similarly by CaMKII at Ser831 (Fig. 7*B*), and by PKA at Ser845 (Fig. 7*C*). Thus, phosphorylation at Ser831/Ser845 is unlikely to be affected by phosphorylation at the MPR, indicating that the effect of Ser816/Ser818 on GluA1 trafficking is independent of the phosphorylation status of Ser 831/Ser845.

### GluA1-MPR regulates heteromeric AMPAR trafficking

**Figure 8:**
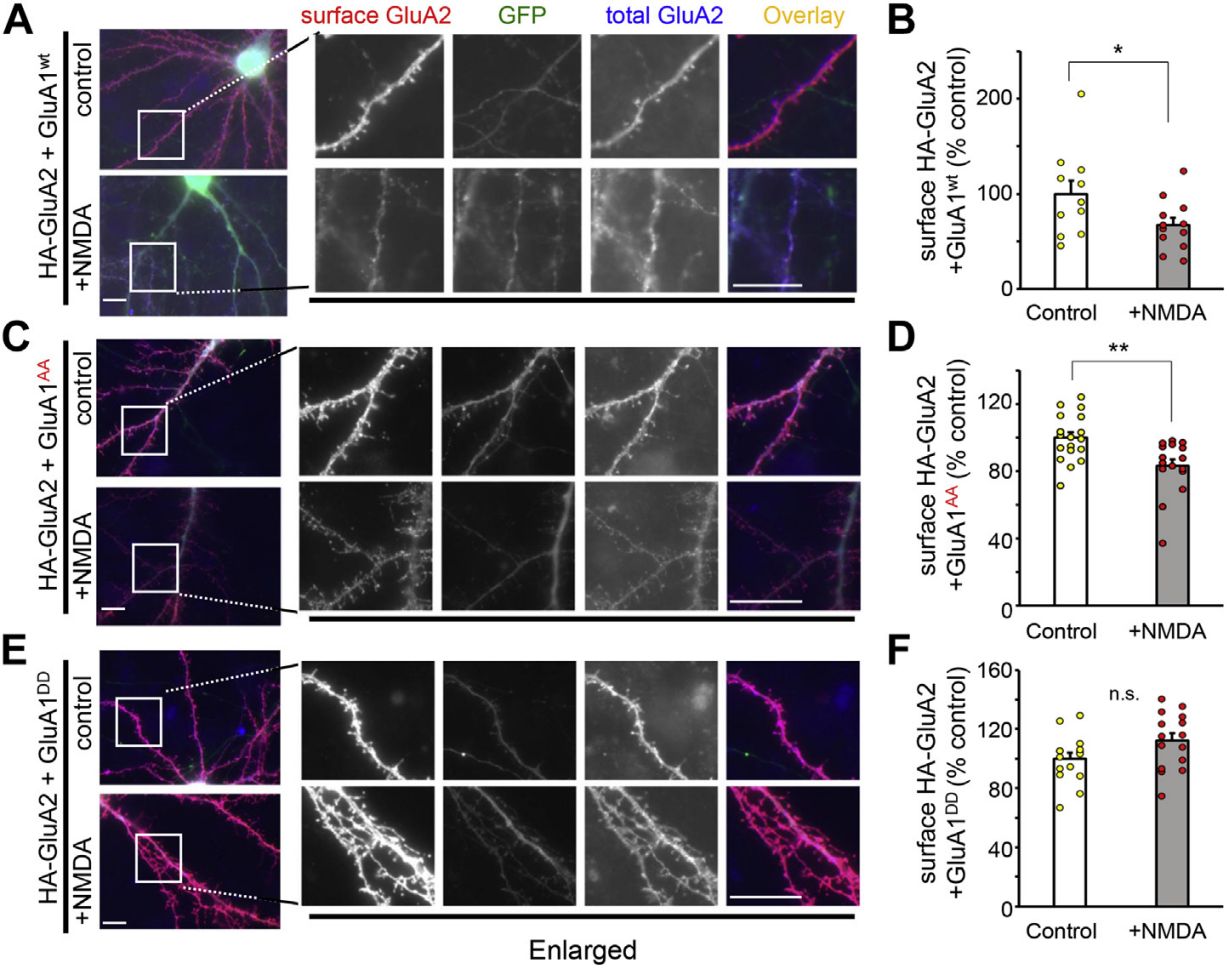
**MPR regulates NMDA-induced trafficking of heteromeric AMPA receptors.***A*–*C*, immunocytochemical analysis of the effects of the MPR on NMDA-induced trafficking of cell surface heteromeric AMPA receptors. Cultured hippocampal neurons expressing WT HA-GluA2 together with GluA1^wt^ (*A*), GluA1^AA^ (*C*), or GluA1^DD^ (*E*) were treated with 50 μM NMDA for 10 min and stained for surface HA-GluA2 (*red*). After Triton X treatment, neurons were stained for total HA-GluA2 (*blue*). The dendritic regions marked by *white squares* were enlarged in the panels to the *right*. The scale bars represent 10 μm. *B*, *D,* and *F*, the graphs represent the quantification of NMDA-induced reduction in the amount of cell surface HA-GluA2 in the presence of GluA1^wt^ or GluA1^AA^ or GluA1^DD^. Data are represented as the ratio of surface HA-GluA2 staining/total HA-GluA2 staining intensity. The ratio in control neurons was defined as 100% (n = 11–19 cells). Data are presented as the mean + SEM and individual data points. ∗∗*p* < 0.01 and ∗*p* < 0.05; n.s. by two-tailed Student’s *t* test. AMPA, AMPA-type glutamate; HA, hemagglutinin; MPR, membrane-proximal region; n.s., not significant; NMDA, N-methyl-d-aspartate.

Endogenous AMPARs mainly exist as diheteromeric GluA1–GluA2 and GluA2–GluA3 receptors in the mammalian brain (22, 23). Next, we expressed HA-tagged GluA2 and untagged GluA1^wt^, GluA1^DD^, or GluA1^AA^ in cultured hippocampal neurons to examine whether the phosphorylation of GluA1-MPR affects the trafficking of heteromeric AMPARs composed of GluA1 and GluA2. After treatment with NMDA (50 μM) for 10 min, the cell surface and total GluA2 were sequentially detected by an anti-HA antibody before and after permeabilizing the plasma membrane. Mutations in the GluA1 MPR did not affect the total and surface expression levels of HA-GluA2 during the basal state (Fig. S3). The intensity of cell surface HA-GluA2 immunoreactivity was significantly reduced by the NMDA treatment in neurons coexpressing GluA1^wt^ (Fig. 8, *A* and *B*; control, 100 ± 14%; NMDA, 67 ± 8%; *p* = 0.045, n = 11–12 cells each), as well as neurons coexpressing GluA1^AA^ (Fig. 8, *C* and *D*; control, 100 ± 3%; NMDA, 83 ± 4%; *p* = 0.002, n = 16–19 cells each), but not in neurons coexpressing GluA1DD (Fig. 8, *E* and *F*; control, 100 ± 4%; NMDA, 112 ± 5%; n = 15 cells each, *p* = 0.07, by two-tailed Student’s *t* test). Because cell-surface HA-GluA2 likely exists in the form of heteromeric receptors with GluA1, these results indicate that the phosphorylation status at the MPR of GluA1 dominantly affects heteromeric AMPAR endocytosis during chemical LTD.

## Discussion

**Figure 9:**
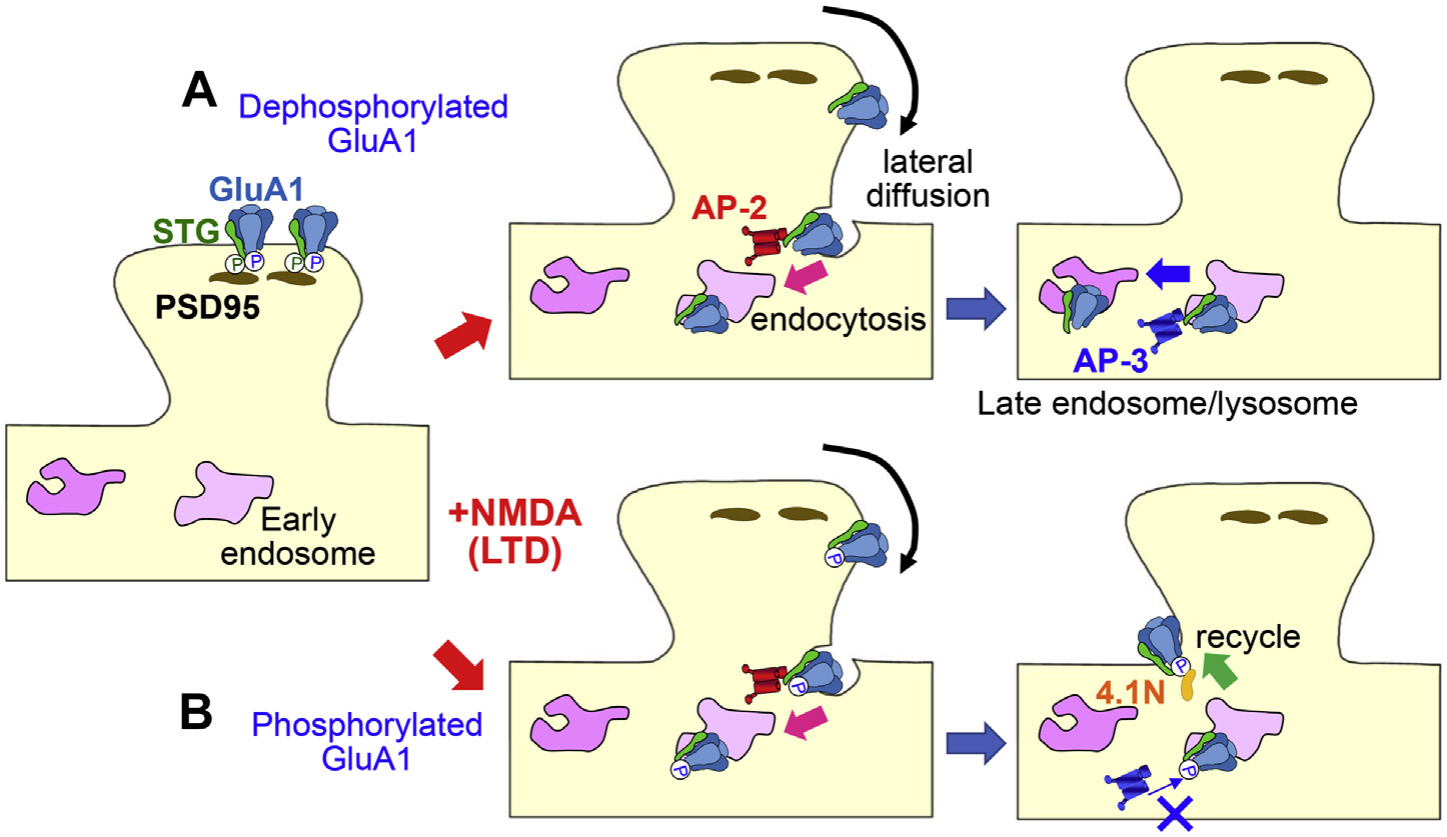
**A model for AMPAR trafficking during LTD achieved by a cross-talk between subunit-dependent and subunit-independent mechanisms.** An auxiliary AMPAR subunit, STG, stabilizes postsynaptic AMPARs by binding to anchoring proteins, such as PSD95. LTD-inducing stimuli dephosphorylate the C terminus of STG and triggers lateral diffusion of the AMPAR–STG complex by reducing the binding affinity of STG to PSD-95. At the endocytic zone, dephosphorylated STG binds to AP-2 to initiate clathrin-dependent endocytosis of the AMPAR–STG complex. In the early endosomes, AP-2 is eventually replaced with AP-3 to facilitate late endosomal/lysosomal trafficking of the AMPAR–STG complex to express LTD (*A*). In contrast, AMPARs containing GluA1 behave differently depending on the phosphorylation status of the MPR, which only occurs in the GluA1 subunit. When the MPR of GluA1 remains phosphorylated, AP-3 cannot be effectively recruited to the AMPAR–STG complex. Such AMPARs are transported back to the cell surface, resulting in impaired LTD (*B*). AMPARs, AMPA-type glutamate receptors; LTD, long-term depression; MPR, membrane-proximal region; PSD95, postsynaptic density 95; STG, stargazin.

It has been unclear whether and how subunit-specific rules of AMPAR trafficking are related to subunit-independent, TARP-mediated AMPAR trafficking mechanisms during LTP/LTD. In the present study, we showed that phosphomimetic mutations of GluA1-MPR inhibited μ3 binding to STG and late endosomal/lysosomal trafficking of AMPARs, which is required for LTD expression (7, 24). Thus, together with earlier findings, we propose a model in which STG-dependent and GluA1-MPR-dependent AMPAR trafficking mechanisms interact with each other during LTD in hippocampal neurons (Fig. 9). At postsynaptic sites, AMPARs are stabilized by anchoring proteins, such as PSD95, which bind to highly phosphorylated STG (17). NMDAR activation induces dephosphorylation of STG (16, 18), releasing the anchor so that the AMPAR–STG complex laterally diffuses to the endocytic zones. At the endocytic zone, AP-2 accumulates (25) and binds to dephosphorylated STG to induce clathrin-mediated endocytosis of the AMPAR–STG complex. In the early endosome, AP-2 is replaced with AP-3 to mediate transport to the late endosomes/lysosomes (Fig. 9*A*). When an AMPAR contains GluA1, in which the MPR remains phosphorylated, AP-3 cannot associate with STG and the AMPAR–STG complex is recycled back to the cell surface by interacting with 4.1N (11, 12) (Fig. 9*B*).

While γ-8 is the dominant TARP in CA1 pyramidal neurons, γ-3 and STG are also modestly expressed (26). Because these TARPs contain conserved serine residues at the C termini that undergo phosphorylation (16), the inhibitory effect of STG mutants on hippocampal LTD may be mediated by the dominant-negative effect of STG. Similarly, normal LTD in γ-8 KO mice (27) may be caused by compensation by the other TARPs (28). Alternatively, STG may play a specific role in the regulation of LTD in CA1 hippocampal neurons because it is highly enriched at perforated synapses (28), which are thought to play an important role in LTD induction (29).

### Hierarchy of AMPAR trafficking mediated by GluA subunits and phosphorylation

Although AMPAR subunits and posttranslational modifications determine the types and extent of synaptic plasticity, a hierarchy may exist such that certain AMPARs are disproportionally recruited to or removed from synapses during LTP and LTD (3). This hierarchy hypothesis explains why LTP (13) and LTD (14) could still be induced in a manner independent of AMPAR subunits. However, it remains unclear how a hierarchy is determined by the subunit-dependent phosphorylation of AMPARs. We postulate that phosphorylation of GluA subunits affects two steps in AMPAR trafficking: anchoring at postsynaptic sites and endocytosis or exocytosis to or from plasma membranes.

For LTD, GluA2 has shown to play a major role in the hierarchy of AMPAR endocytosis in many brain regions (3). Specifically, phosphorylation of GluA2 Ser880 regulates LTD in the cerebellum (8) and the hippocampus (30). This effect is likely explained by the anchoring of GluA2-containing AMPARs by GRIP1/2 and PICK1 (31, 32). Phosphorylation at Ser880 by PKC releases GluA2 from the GRIP1/2 anchor during cerebellar LTD (33, 34). However, surface AMPARs are tightly associated with TARPs, through which the AMPAR–TARP complex is anchored to postsynaptic sites. Thus, the release from GRIP could not fully explain the dominant role of GluA2 during LTD.

At the endocytic zone, AMPARs need to be recognized by μ2 for clathrin-dependent endocytosis. Although the MPR of GluA2 was shown to bind to the μ2 subunit of AP-2 (35), μ2 is mainly recruited to AMPARs by binding to dephosphorylated STG in a manner independent of GluA subunits (18) and their phosphorylation status (Fig. 1). GluA2, in which Ser880 is phosphorylated, could bind to PICK1 at the endocytic zone, which has been shown to recruit the α subunit of AP-2 and dynamin (36). Thus, the dominant role of GluA2 in LTD could be partly attributed to its preferential binding to PICK1.

After endocytosis, AMPARs need to be trafficked to late endosomes/lysosomes for LTD expression (7, 24). Unlike μ2, the μ3 subunit of AP-3 could not be recruited to STG unless the MPR of GluA1 was fully dephosphorylated (Fig. 8*B*). Thus, the absence of phosphorylation sites at the MPR of GluA2 (Fig. 1*A*) could also contribute to the preferential role of GluA2-containing and GluA1-lacking AMPARs in LTD expression.

Phosphorylation of the MPR of GluA1 by PKC was previously shown to promote synaptic incorporation of AMPARs during LTP (11, 12). Similarly, GluA1, which contained phosphomimetic mutations in the MPR, was recycled from the endosome to the cell surface (Fig. 5). Because AMPARs are reported to be exocytosed from recycling endosomes (37), phosphorylation-dependent binding to μ3 by the MPR of GluA1 may also explain the subunit-selective hierarchy in LTP expression.

### Toward a unified theory of AMPAR trafficking

There remain many questions about how other phosphorylation sites of GluA subunits affect the hierarchy of AMPAR trafficking. For example, although phosphorylation at Ser845 of GluA1 is required for LTD induction (9, 10), the mechanisms by which such subunit-specific phosphorylation affects LTD is achieved remain unclear. Recently, phosphorylation at Ser845 was shown to transiently recruit GluA1-containing, Ca^2+^-permeable AMPARs to postsynaptic sites to fully activate calcineurin during LTD (38). Indeed, calcineurin is absolutely required to dephosphorylate TARP to release the AMPAR–TARP complex from the postsynaptic anchor during hippocampal and cerebellar LTD (16, 19). However, it is unclear how phosphorylation at Ser845 mediates preferential trafficking of GluA1 to postsynaptic sites. Similarly, the mechanisms by which phosphorylation at Ser831 of GluA1 contribute to LTP remain unclear. Although phosphomimetic and phospho-deficient mutations at the MPR did not affect the phosphorylation at Ser831/Ser845 (Fig. 8, *B* and *C*), phosphorylation at Ser831/Ser845 was shown to work in concert with Ser818 phosphorylation to trigger the stable incorporation of GluA1 during hippocampal LTP (11). Thus, the effect of phosphorylation at Ser831 and Ser845 on AMPAR trafficking could be partly attributed to phosphorylation levels at the MPR, which determine the association with AP-3 and 4.1N.

In addition to regulating AMPAR trafficking, phosphorylation of the GluA1 C termini may contribute to LTP/LTD by regulating the channel conductance and the heteromeric assembly of AMPARs. Because the phosphomimetic and phospho-deficient mutations at Ser818 similarly prevented AKAP79-induced increase in GluA1 homomers (39), this effect will not be involved in GluA1 phosphomimetic status-dependent AMPAR trafficking during LTD. On the other hand, PKC phosphorylation at Ser818 increases the channel conductance of AMPARs (40). Thus, the dephosphorylation at Ser818 may enhance LTD induction by decreasing the channel conductance of the synaptic AMPA receptors in addition to the reduction in the number of cell-surface AMPA receptors.

Because differential phosphorylation of AMPARs is reported in certain mouse models of neuropsychiatric disease, such as fragile X mental retardation (41), further studies are warranted to clarify the molecular mechanisms by which phosphorylation and other posttranslational modifications regulate the hierarchy of AMPAR trafficking.

## Experimental procedures

### Mice

All procedures related to animal care and treatment were performed in accordance with the guidelines approved by the animal resource committees of the University of Electro-Communications and Keio University. Mice were housed with a 12:12 h light–dark cycle with food and water available *ad libitum*.

### Chemicals and antibodies

NMDA was purchased from Tocris Bioscience. Commercial antibodies were as follows: anti-GluA1 (04-855, Millipore), anti-GluA1 (SAB5201086, Sigma), anti phospho-GluA1 (Ser831) (36-8200, Invitrogen), anti-phospho-GluA1 (Ser845) (36-8300, Invitrogen), anti-stargazin (C8206, Sigma), anti-4.1N (276103, Synaptic Systems), anti-GST (RPN1236, Amersham), anti-HA, (901501, Covance), anti-FLAG (F7425, Sigma), and anti-MAP-2 (AB5622, Millipore) antibodies; Alexa 350 (A-11045, Thermo Fisher), Alexa 405 (A-31556, Thermo Fisher), Alexa 488 (A-11008, Thermo Fisher), Alexa 546 (A-11003, Thermo Fisher), HRP (18-8816-33, 18-8817-33, Rockland) conjugated secondary antibodies, and pre-immune IgG (CYP450-GP HU-A000).

### Construction and transfection or transformation of expression plasmids

Using a PCR method and Pyrobest (Takara), the serine residues encoding Ser816, Ser818, Ser831, and Ser845 in mouse GluA1 cDNA were mutated to encode aspartate or alanine. The cDNA-encoding HA was added to the 5' end (immediately following the signal sequence) of mutant GluA1 and WT GluA2. The cDNA-encoding FLAG-tag was added to the 3′ end (immediately upstream of the stop codon) of mouse μ2 or mouse μ3A cDNAs. The nucleotide sequences of the amplified ORFs were confirmed by bidirectional sequencing. After the cDNAs were cloned into the expression vectors, either pTracer (Invitrogen) or pCAGGS (provided by Dr J Miyazaki, Osaka University, Osaka, Japan), the constructs were transfected into human embryonic kidney 293T (HEK293T) cells using the Ca^2+^-phosphate method or were transfected into cultured hippocampal neurons using Lipofectamine 2000 (Invitrogen).

For the expression of GST-fusion protein, the cDNA encoding the C-terminal region of WT or mutant TARPs or GluA1 was amplified by PCR and cloned into pGEX 4T-2. *Escherichia coli* BL21(DE3) was transformed by pGEX expression vectors and grown in 100 ml of LB medium. The expression of GST fusion proteins was induced by the addition of IPTG 0.1 mM. BL21(DE3) cells were disrupted by sonication in 10 ml of PBS, and 500 μl of Glutathione Sepharose column (Amersham Pharmacia) suspension was added to the supernatant. After washing with 1 ml PBS five times, GST fusion proteins were eluted with 1 ml of the elution buffer (100 mM Tris HCl, 10 mM glutathione, pH 8.0).

### Culture of hippocampal neuron

Hippocampi dissected from E16/17 ICR mice were treated with 10 U ml^−1^ trypsin and 100 U ml^−1^ DNase in Dulbecco's modified Eagle's medium at 37 °C for 20 min. The dissociated hippocampal neurons were plated on PEI-coated glass coverslips and cultured in Neurobasal medium (Invitrogen) with B-27 (Gibco) or NS21 supplement (42) and 0.5 mM L-glutamine. After 7 to 10 days in vitro culture, neurons were transiently transfected with plasmids using Lipofectamine 2000 and used for the AMPA receptor endocytosis or recycling assays.

### Assay for AMPA receptor endocytosis

Hippocampal neurons transfected with pCAGGS expression vectors for mutant HA-GluA1 plus GFP or WT HA-GluA2 plus mutant GluA1 were stimulated with 50 μM NMDA for 10 min and fixed in 4% paraformaldehyde without permeabilization, for 10 min at room temperature (RT). After fixed neurons were washed with PBS and incubated with a blocking solution (2% BSA and 2% normal goat serum in PBS), surface HA-GluA1 or HA-GluA2 were labeled with the anti-HA antibody (1:1000) and visualized with Alexa 546 secondary antibody (1:1000). To label total HA-GluA1 or HA-GluA2, neurons were permeabilized and blocked with a blocking solution containing 0.4% Triton X-100 and incubated with the anti-HA antibody (1:1000) and Alexa 350 secondary antibodies (1:1000). Fluorescence images were captured using a fluorescence microscope (BX60, Olympus) equipped with a CCD camera (DP 70, Olympus) and analyzed using IPLab software (Scanalytics). For statistical analysis of the surface expression level of HA-GluA1 or HA-GluA2, the intensity of Alexa 546 for surface HA-GluA1 or HA-GluA2 was measured and normalized using the intensity of Alexa 350 for total HA-GluA1 or HA-GluA2. The fluorescence intensity on the dendrites at least 20 μm away from the soma was measured. In the representative images, brightness and contrast were adjusted uniformly within each experimental series for consistent visibility.

### Assay for AMPA receptor recycling

Recycling of AMPA receptors was analyzed by the method described by Nooh *et al.* (43). Living hippocampal neurons transfected with plasmids for mutant HA-GluA1 were labeled with the anti-HA antibody (1:100) for 1 h. After washing out the excess amount of antibody, neurons were stimulated with 50 μM NMDA for 3 min. After washing out the NMDA, neurons were treated with 0.5 M NaCl and 0.2 M acetic acid for 4 min at 0 °C. After washing out NaCl and acetic acid, neurons were incubated for 30 min at 37 °C in a neurobasal medium with B27 supplement. The neurons were then fixed in 4% paraformaldehyde without permeabilization, for 10 min at RT. After fixed neurons were washed with PBS and incubated in a blocking solution (2% BSA and 2% normal goat serum in PBS), the surface HA antibody was visualized with Alexa 546 secondary antibody (1:1000). To label internalized HA-GluA1, neurons were permeabilized and blocked with the blocking solution containing 0.4% Triton X-100 and incubated with the Alexa 350 secondary antibodies (1:1000). Fluorescence images were captured by a fluorescence microscope equipped with a CCD camera and analyzed using IPLab software. For statistical analysis of the recycled HA-GluA1, the intensity of Alexa 546 for recycled HA-GluA1 was measured and normalized using the intensity of Alexa 350 for internalized HA-tagged GluA1. The fluorescence intensity on the dendrites at least 20 μm away from the soma was measured. In the representative images, brightness and contrast were adjusted uniformly within each experimental series for consistent visibility.

### Colocalization assay of HA-GluA1 and Rab proteins

Hippocampal neurons transfected with pCAGGS expression vectors for mutant HA-GluA1, Rab4, or Rab7-EGFP were stimulated with 50 μM NMDA for 3 or 10 min and fixed in 4% paraformaldehyde. After fixed neurons were washed with PBS and incubated with a blocking solution (2% BSA and 2% normal goat serum 0.4% Triton-X in PBS), the neurons were incubated with the anti-HA antibody (1:1000) and anti-MAP-2 antibody (1:1000) for 1 h at RT. After washing with PBS, neurons were incubated with Alexa 546 and Alexa 405 secondary antibodies (1:1000; Invitrogen). Fluorescence images were captured using a confocal microscope (FV1200, Olympus) and analyzed using IPLab software (Scanalytics). To statistically analyze the colocalization of the HA-GluA1 and Rab proteins, the intensities of Alexa 546 on the EGFP-positive regions were measured and normalized using the total intensity of Alexa 546. The fluorescence intensity on the dendrites at least 20 μm away from the soma was measured. In the representative images, brightness and contrast were adjusted uniformly within each experimental series for consistent visibility.

### *In vitro* phosphorylation of GST-GluA1CT

Purified GST fusion proteins (20 μl) with a GluA1 C terminus were subjected to an *in vitro* phosphorylation assay using the CAMK2a Kinase Enzyme System and PKA Kinase Enzyme System according to the manufacturer’s protocol (Promega). Phosphorylated GST fusion proteins were analyzed by immunoblot analysis using anti-Phospho-GluA1 (Ser831), Phospho-GluA1 (Ser845) (Invitrogen), and anti-GST (Amersham) antibodies.

### Immunoprecipitation, pull-down assay, and immunoblot assays

Transfected HEK293T cells were solubilized in 6-cm dishes in 500 μl of TNE buffer (50 mM NaCl, 10% NP-40, 20 mM EDTA, 0.1% SDS, 50 mM Tris HCl, pH 8.0) supplemented with a protease inhibitor cocktail (Calbiochem). Cultured hippocampal neurons (days in vitro 17) from three wells of the 12-well dish (Falcon) were solubilized in 300 μl of the lysis buffer (250 mM NaCl, 1.5% Triton-X, 5 mM EDTA, and 25 mM Tris HCl, pH 7.4), with a protease inhibitor cocktail. Finally, 0.5% of the total lysate was applied to the immunoblot analysis as the input.

For the immunoprecipitation assays, 5 μl of anti-GluA1 (Millipore) or anti 4.1N (Synaptic Systems) or preimmune IgG (CYP450-GP) was added to the samples, and the mixture was incubated for 1 h at 4 °C. Then, 50 μl of protein G-conjugated agarose (Amersham) was added, and this mixture was incubated for 1 h at 4 °C. After the precipitates were washed four times with 500 μl of TNE buffer or lysis buffer, 50 μl of SDS-PAGE sample buffer was added and the samples were incubated for 5 min at 95 °C. After centrifugation, 5 μl of the supernatant was analyzed using immunoblotting with anti-FLAG (Sigma), anti-GluA1 (Sigma), anti-stargazin (Sigma), and anti-4.1N (Synaptic Systems) antibodies, TrueBlot HRP-conjugated secondary antibody (Rockland), and the Immobilon Western kit (Millipore). The chemiluminescence signals were detected by LuminoGraph II (ATTO) and quantified using CS Analyzer software (ATTO).

For GST pull-down assays, purified GST fusion proteins (50 μl) with a TARP C terminus were incubated with the lysate of HEK293T cells expressing the μ subunit of AP in the presence or absence of 500 μM of peptides corresponding to the MPR of AMPA receptors. After a 1-h incubation at 4 °C, GST proteins were pulled down by glutathione Sepharose resins (Amersham). About 50 μl of SDS-PAGE sample buffer was added to the precipitates and the samples were incubated for 5 min at 95 °C. After centrifugation, 5 μl of the supernatant was analyzed by immunoblot analysis with anti-FLAG (Sigma) and anti GST (Amersham) antibodies.

For the biotinylated peptide (EFCYKSRSES KRMK) pull-down assay of GST fusion proteins, 50 μl of purified GST fusion proteins with a TARP C terminus was incubated with the biotinylated peptide corresponding to the MPR of AMPA receptors (500 μM) in 500 μl of PBS. For the biotinylated peptide pull-down assay of FLAG-μ3, HEK293 cells expressing FLAG μ3 were solubilized in 500 μl TNE, and 500 μM biotinylated peptide was added together with 5 μg of GST or GST-STG fusion proteins. After incubation at 4 °C for 1 h, biotinylated peptides were pulled down using 50 μl of streptavidin-conjugated magnetic beads (Invitrogen), and the precipitates were analyzed by immunoblot analysis.

In the representative images, brightness and contrast were adjusted uniformly within each experimental series for consistent visibility.

## Data availability

All data described are presented either within the article or in the supporting information.

## Supporting information

This article contains supporting information.

## Conflict of interest

The authors declare that they have no conflicts of interest with the contents of this article.

## References

[1] Collingridge G.L., Peineau S., Howland J.G., Wang Y.T. (2010). Long-term depression in the CNS. Nat. Rev. Neurosci..

[2] Nicoll R.A. (2017). A brief history of long-term potentiation. Neuron.

[3] Diering G.H., Huganir R.L. (2018). The AMPA receptor code of synaptic plasticity. Neuron.

[4] Groc L., Choquet D. (2020). Linking glutamate receptor movements and synapse function. Science.

[5] Esteban J.A., Shi S.H., Wilson C., Nuriya M., Huganir R.L., Malinow R. (2003). PKA phosphorylation of AMPA receptor subunits controls synaptic trafficking underlying plasticity. Nat. Neurosci..

[6] Shi S., Hayashi Y., Esteban J.A., Malinow R. (2001). Subunit-specific rules governing AMPA receptor trafficking to synapses in hippocampal pyramidal neurons. Cell.

[7] Lee S.H., Simonetta A., Sheng M. (2004). Subunit rules governing the sorting of internalized AMPA receptors in hippocampal neurons. Neuron.

[8] Chung H.J., Steinberg J.P., Huganir R.L., Linden D.J. (2003). Requirement of AMPA receptor GluR2 phosphorylation for cerebellar long-term depression. Science.

[9] Lee H.K., Barbarosie M., Kameyama K., Bear M.F., Huganir R.L. (2000). Regulation of distinct AMPA receptor phosphorylation sites during bidirectional synaptic plasticity. Nature.

[10] Lee H.K., Takamiya K., Han J.S., Man H., Kim C.H., Rumbaugh G., Yu S., Ding L., He C., Petralia R.S., Wenthold R.J., Gallagher M., Huganir R.L. (2003). Phosphorylation of the AMPA receptor GluR1 subunit is required for synaptic plasticity and retention of spatial memory. Cell.

[11] Boehm J., Kang M.G., Johnson R.C., Esteban J., Huganir R.L., Malinow R. (2006). Synaptic incorporation of AMPA receptors during LTP is controlled by a PKC phosphorylation site on GluR1. Neuron.

[12] Lin D.T., Makino Y., Sharma K., Hayashi T., Neve R., Takamiya K., Huganir R.L. (2009). Regulation of AMPA receptor extrasynaptic insertion by 4.1N, phosphorylation and palmitoylation. Nat. Neurosci..

[13] Granger A.J., Shi Y., Lu W., Cerpas M., Nicoll R.A. (2013). LTP requires a reserve pool of glutamate receptors independent of subunit type. Nature.

[14] Granger A.J., Nicoll R.A. (2014). LTD expression is independent of glutamate receptor subtype. Front. Synaptic Neurosci..

[15] Nicoll R.A., Tomita S., Bredt D.S. (2006). Auxiliary subunits assist AMPA-type glutamate receptors. Science.

[16] Tomita S., Stein V., Stocker T.J., Nicoll R.A., Bredt D.S. (2005). Bidirectional synaptic plasticity regulated by phosphorylation of stargazin-like TARPs. Neuron.

[17] Sumioka A., Yan D., Tomita S. (2010). TARP phosphorylation regulates synaptic AMPA receptors through lipid bilayers. Neuron.

[18] Matsuda S., Kakegawa W., Budisantoso T., Nomura T., Kohda K., Yuzaki M. (2013). Stargazin regulates AMPA receptor trafficking through adaptor protein complexes during long-term depression. Nat. Commun..

[19] Nomura T., Kakegawa W., Matsuda S., Kohda K., Nishiyama J., Takahashi T., Yuzaki M. (2012). Cerebellar long-term depression requires dephosphorylation of TARP in Purkinje cells. Eur. J. Neurosci..

[20] Zhou Z., Liu A., Xia S., Leung C., Qi J., Meng Y., Xie W., Park P., Collingridge G.L., Jia Z. (2018). The C-terminal tails of endogenous GluA1 and GluA2 differentially contribute to hippocampal synaptic plasticity and learning. Nat. Neurosci..

[21] Lu W., Roche K.W. (2012). Posttranslational regulation of AMPA receptor trafficking and function. Curr. Opin. Neurobiol..

[22] Lu W., Shi Y., Jackson A.C., Bjorgan K., During M.J., Sprengel R., Seeburg P.H., Nicoll R.A. (2009). Subunit composition of synaptic AMPA receptors revealed by a single-cell genetic approach. Neuron.

[23] Zhao Y., Chen S., Swensen A.C., Qian W.J., Gouaux E. (2019). Architecture and subunit arrangement of native AMPA receptors elucidated by cryo-EM. Science.

[24] Fernandez-Monreal M., Brown T.C., Royo M., Esteban J.A. (2012). The balance between receptor recycling and trafficking toward lysosomes determines synaptic strength during long-term depression. J. Neurosci..

[25] Unoki T., Matsuda S., Kakegawa W., Van N.T., Kohda K., Suzuki A., Funakoshi Y., Hasegawa H., Yuzaki M., Kanaho Y. (2012). NMDA receptor-mediated PIP5K activation to produce PI(4,5)P(2) is essential for AMPA receptor endocytosis during LTD. Neuron.

[26] Tomita S., Chen L., Kawasaki Y., Petralia R.S., Wenthold R.J., Nicoll R.A., Bredt D.S. (2003). Functional studies and distribution define a family of transmembrane AMPA receptor regulatory proteins. J. Cell Biol..

[27] Rouach N., Byrd K., Petralia R.S., Elias G.M., Adesnik H., Tomita S., Karimzadegan S., Kealey C., Bredt D.S., Nicoll R.A. (2005). TARP gamma-8 controls hippocampal AMPA receptor number, distribution and synaptic plasticity. Nat. Neurosci..

[28] Yamasaki M., Fukaya M., Yamazaki M., Azechi H., Natsume R., Abe M., Sakimura K., Watanabe M. (2016). TARP gamma-2 and gamma-8 differentially control AMPAR density across Schaffer collateral/commissural synapses in the hippocampal CA1 area. J. Neurosci..

[29] Luscher C., Nicoll R.A., Malenka R.C., Muller D. (2000). Synaptic plasticity and dynamic modulation of the postsynaptic membrane. Nat. Neurosci..

[30] Seidenman K.J., Steinberg J.P., Huganir R., Malinow R. (2003). Glutamate receptor subunit 2 serine 880 phosphorylation modulates synaptic transmission and mediates plasticity in CA1 pyramidal cells. J. Neurosci..

[31] Steinberg J.P., Takamiya K., Shen Y., Xia J., Rubio M.E., Yu S., Jin W., Thomas G.M., Linden D.J., Huganir R.L. (2006). Targeted *in vivo* mutations of the AMPA receptor subunit GluR2 and its interacting protein PICK1 eliminate cerebellar long-term depression. Neuron.

[32] Takamiya K., Mao L., Huganir R.L., Linden D.J. (2008). The glutamate receptor-interacting protein family of GluR2-binding proteins is required for long-term synaptic depression expression in cerebellar Purkinje cells. J. Neurosci..

[33] Matsuda S., Launey T., Mikawa S., Hirai H. (2000). Disruption of AMPA receptor GluR2 clusters following long-term depression induction in cerebellar Purkinje neurons. EMBO J..

[34] Xia J., Chung H.J., Wihler C., Huganir R.L., Linden D.J. (2000). Cerebellar long-term depression requires PKC-regulated interactions between GluR2/3 and PDZ domain-containing proteins. Neuron.

[35] Kastning K., Kukhtina V., Kittler J.T., Chen G., Pechstein A., Enders S., Lee S.H., Sheng M., Yan Z., Haucke V. (2007). Molecular determinants for the interaction between AMPA receptors and the clathrin adaptor complex AP-2. Proc. Natl. Acad. Sci. U. S. A..

[36] Fiuza M., Rostosky C.M., Parkinson G.T., Bygrave A.M., Halemani N., Baptista M., Milosevic I., Hanley J.G. (2017). PICK1 regulates AMPA receptor endocytosis via direct interactions with AP2 alpha-appendage and dynamin. J. Cell Biol..

[37] Park M., Penick E.C., Edwards J.G., Kauer J.A., Ehlers M.D. (2004). Recycling endosomes supply AMPA receptors for LTP. Science.

[38] Sanderson J.L., Gorski J.A., Dell'Acqua M.L. (2016). NMDA receptor-dependent LTD requires transient synaptic incorporation of Ca(2)(+)-permeable AMPARs mediated by AKAP150-anchored PKA and calcineurin. Neuron.

[39] Summers K.C., Bogard A.S., Tavalin S.J. (2019). Preferential generation of Ca(2+)-permeable AMPA receptors by AKAP79-anchored protein kinase C proceeds via GluA1 subunit phosphorylation at Ser-831. J. Biol. Chem..

[40] Jenkins M.A., Wells G., Bachman J., Snyder J.P., Jenkins A., Huganir R.L., Oswald R.E., Traynelis S.F. (2014). Regulation of GluA1 alpha-amino-3-hydroxy-5-methyl-4-isoxazolepropionic acid receptor function by protein kinase C at serine-818 and threonine-840. Mol. Pharmacol..

[41] Tian M., Zeng Y., Hu Y., Yuan X., Liu S., Li J., Lu P., Sun Y., Gao L., Fu D., Li Y., Wang S., McClintock S.M. (2015). 7, 8-Dihydroxyflavone induces synapse expression of AMPA GluA1 and ameliorates cognitive and spine abnormalities in a mouse model of fragile X syndrome. Neuropharmacology.

[42] Chen Y., Stevens B., Chang J., Milbrandt J., Barres B.A., Hell J.W. (2008). NS21: Re-defined and modified supplement B27 for neuronal cultures. J. Neurosci. Methods.

[43] Nooh M.M., Chumpia M.M., Hamilton T.B., Bahouth S.W. (2014). Sorting of beta1-adrenergic receptors is mediated by pathways that are either dependent on or independent of type I PDZ, protein kinase A (PKA), and SAP97. J. Biol. Chem..

