# Supplementary material for: Subunit-dependent and subunit-independent rules of AMPA receptor trafficking during chemical long-term depression in hippocampal neurons
Source: J Biol Chem. 2021 Jul 10;297(2):100949. doi: 10.1016/j.jbc.2021.100949 (PMC8335659; doi:10.1016/j.jbc.2021.100949)
Supplement: Supplemental Figures S1–S3 [file mmc1.pdf]

Supporting Information

Subunit-dependent and independent rules of AMPA receptor trafficking during chemical long-term depression in hippocampal neurons

Shinji Matsuda[1,2,3], Michisuke Yuzaki[3]

[1]Department of Engineering Science, Graduate School of Informatics and Engineering; [2]Center for Neuroscience and Biomedical Engineering (CNBE), The University of Electro-Communications, Tokyo 182-8585, Japan; [3]Department of Physiology, Keio University School of Medicine, Tokyo 160-8582, Japan

Table of contents:

Figure S1: Immunoprecipitation by pre-immune IgG
Figure S2: Total and surface expression levels of GluA1 at the basal state
Figure S3: Total and surface expression levels of GluA2 at the basal state

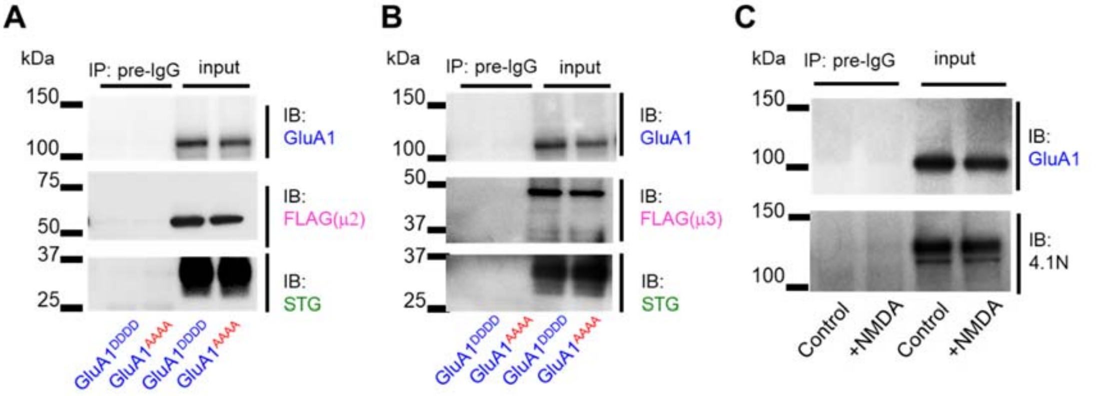

Figure S1. Immunoprecipitation by pre-immune IgG. **A, B**, Cell lysates of HEK293T cells expressing mutant GluA1, STG, and FLAG-tagged μ2 (A) or μ3 (B) were subjected to immunoprecipitation using pre-immune IgG and analyzed by immunoblot analysis. GluA1, STG, μ2, and μ3 were not detected in the immunoprecipitated fractions. **C.** Cultured hippocampal neurons underwent immunoprecipitation using pre-immune IgG and were analyzed by immunoblot analysis. GluA1 and 4.1N were not detected in the immunoprecipitate fractions.

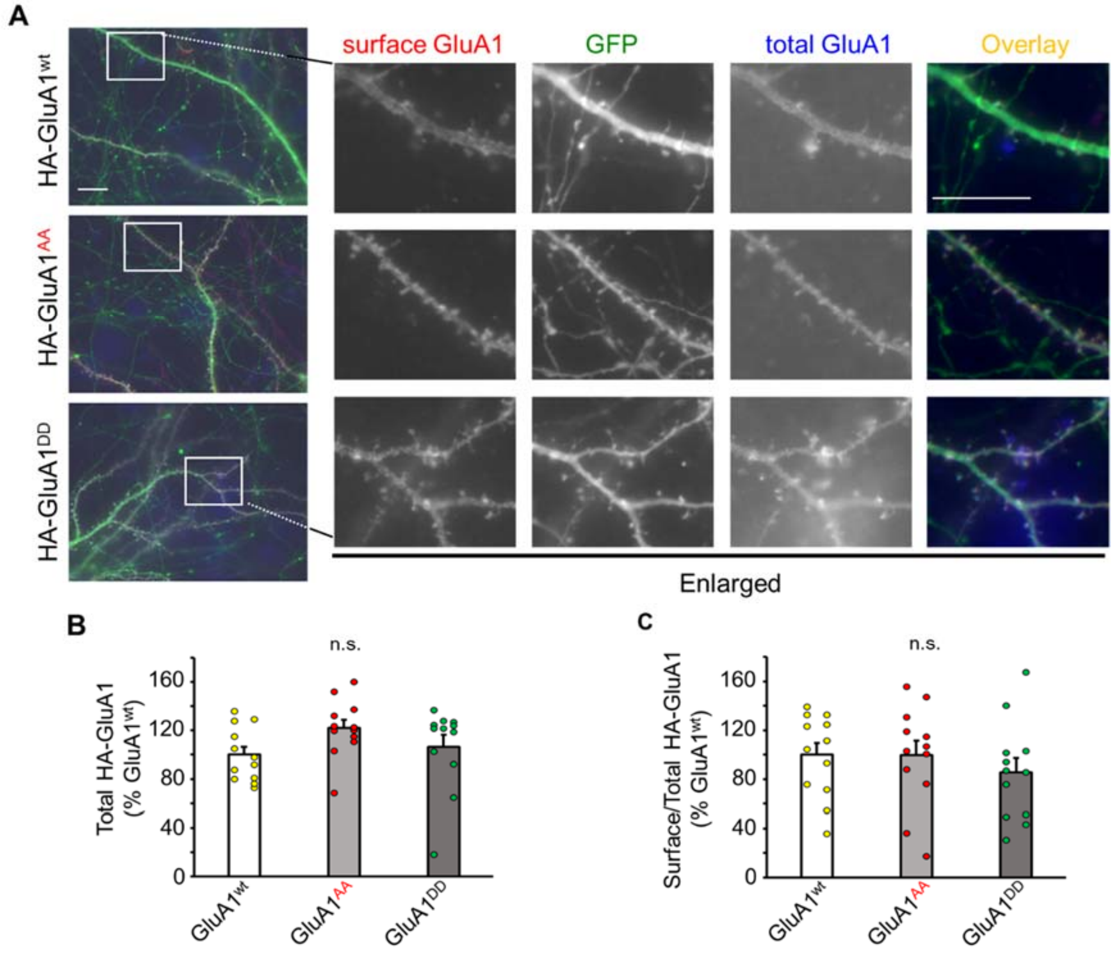

Figure S2. Mutations in the MPR did not affect the total and surface expression levels of GluA1 at the basal state. **A.** Immunocytochemical analysis of the effects of MPR mutations on total and surface GluA1 levels at the basal state. Cultured hippocampal neurons expressing EGFP and HA-tagged wild type (top, GluA1^{wt}) or phospho-deficient GluA1 (center, GluA1^{AA}) or phosphomimetic GluA1 (bottom, GluA1^{DD}) were fixed. HA-GluA1 was detected before (surface, red) and after (total, blue) treatment with Triton-X. The dendritic regions marked by squares are enlarged in the panels on the right (scale bars, 10 μm). **B, C.** Quantification of the basal expression level (**B**) and the ratio of the surface to total HA-GluA1 (**C**) without NMDA treatment. The mean fluorescence intensity of the total HA-GluA1 (**B**) and the ratio of the surface to total HA-GluA1 (**C**) in neurons expressing HA-GluA1^{wt} were defined as 100% (n = 8-14). The data are presented as mean + SEM and individual data points (n.s., not significant by one-way ANOVA).

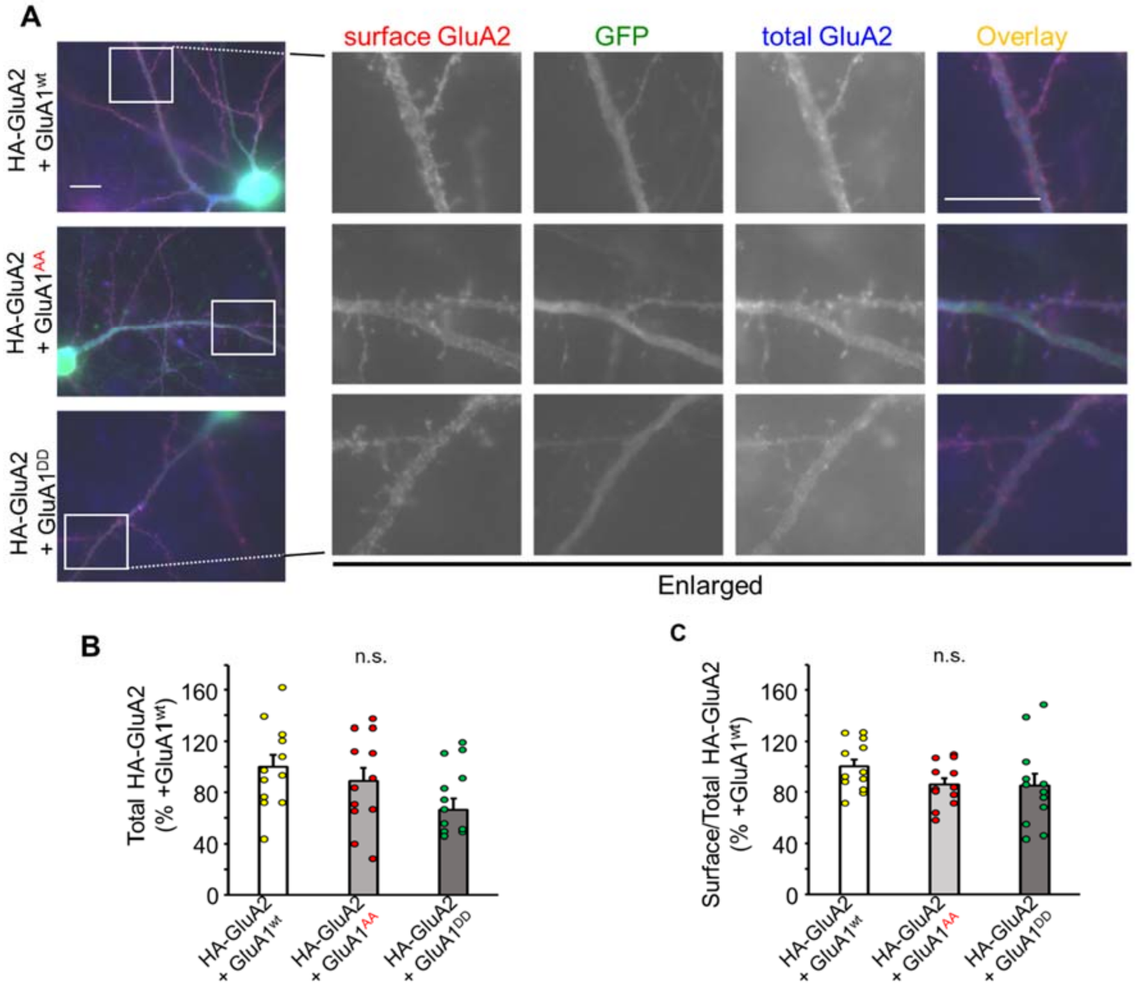

Figure S3. Mutations in the GluA1 MPR did not affect total and surface expression levels of heteromeric AMPA receptors. **A.** Immunocytochemical analysis of the effects of GluA1 MPR mutations on total and cell surface HA-GluA2. Cultured hippocampal neurons expressing EGFP and HA-tagged wild type GluA2 along with wild type GluA1 (top, $GluA1^{wt}$), or phospho-deficient GluA1 (center, $GluA1^{AA}$), or phosphomimetic GluA1 (bottom, $GluA1^{DD}$) were fixed. HA-GluA2 was detected before (surface, red) and after (total, blue) treatment with Triton-X. The dendritic regions marked by squares are enlarged in the panels on the right (scale bars, 10 µm). **B, C.** Quantification of the basal expression level (**B**) and the ratio of the surface to total HA-GluA2 (**C**) without NMDA treatment. The mean fluorescence intensity of total HA-GluA2 (**B**) and the ratio of surface to total HA-GluA2 (**C**) in neurons co-expressing $GluA1^{wt}$ were defined as 100% (n = 8−14). Data are presented as mean + SEM and individual data points (n.s., not significant by one-way ANOVA).
